# Integrative Taxonomy Reveals Hidden Cryptic Diversity within Pin Nematodes of the Genus *Paratylenchus* (Nematoda: Tylenchulidae)

**DOI:** 10.3390/plants10071454

**Published:** 2021-07-15

**Authors:** Ilenia Clavero-Camacho, Juan Emilio Palomares-Rius, Carolina Cantalapiedra-Navarrete, Guillermo León-Ropero, Jorge Martín-Barbarroja, Antonio Archidona-Yuste, Pablo Castillo

**Affiliations:** 1 Instituto de Agricultura Sostenible (IAS), Consejo Superior de Investigaciones Científicas (CSIC), Avenida Menéndez Pidal s/n, Campus de Excelencia Internacional Agroalimentario, ceiA3, 14004 Córdoba, Spain; iclavero@ias.csic.es (I.C.-C.); palomaresje@ias.csic.es (J.E.P.-R.); ccantalapiedra@ias.csic.es (C.C.-N.); gleon@ias.csic.es (G.L.-R.); jorgemb@ias.csic.es (J.M.-B.); 2 Andalusian Institute of Agricultural and Fisheries Research and Training (IFAPA), Centro Alameda del Obispo, 14004 Córdoba, Spain; antonio.archidona-yuste@ufz.de; 3 Department of Ecological Modelling, Helmholtz Centre for Environmental Research—UFZ, Permoserstrasse 15, 04318 Leipzig, Germany

**Keywords:** cytochrome c oxidase subunit 1, ITS rRNA, D2-D3 of 28S rRNA, molecular, morphology, phylogeny, rRNA, taxonomy

## Abstract

This study delves into the diagnosis of pin nematodes (*Paratylenchus* spp.) in Spain based on integrative taxonomical approaches using 24 isolates from diverse natural and cultivated environments. Eighteen species were identified using females, males (when available) and juveniles with detailed morphology-morphometry and molecular markers (D2-D3, ITS and COI). Molecular markers were obtained from the same individuals used for morphological and morphometric analyses. The cryptic diversity using an integrative taxonomical approach of the *Paratylenchus straeleni*-species complex was studied, consisting of an outstanding example of the cryptic diversity within *Paratylenchus* and including the description of a new species, *Paratylenchus parastraeleni* sp. nov. Additionally, 17 already known species were identified comprising *P. amundseni*, *P. aciculus*, *P. baldaccii*, *P. enigmaticus*, *P. goodeyi*, *P. holdemani*, *P. macrodorus*, *P. neoamblycephalus*, *P. pandatus*, *P. pedrami*, *P. recisus*, *P. sheri*, *P. tateae*, *P. variabilis*, *P. veruculatus*, *P. verus*, and *P. vitecus*. Eight of these species need to be considered as first reports for Spain in this work (*viz*. *P. amundseni*, *P. aciculus*, *P. neoamblycephalus*, *P. pandatus*, *P. recisus, P. variabilis, P. verus* and *P. vitecus*). Thirty-nine species of *Paratylenchus* have been reported in Spain from cultivated and natural ecosystems. Although we are aware that nematological efforts on *Paratylenchus* species in Southern Spain have been higher than that carried out in central and northern part of the country, the present distribution of the genus in Spain, with about 90% of species (35 out of 39 species, and 24 of them confirmed by integrative taxonomy) only reported in Southern Spain, suggest that this part of the country can be considered as a potential hotspot of biodiversity.

## 1. Introduction

Pin nematodes of the genus *Paratylenchus* Micoletzky, 1922 [1] are obligate plant-ectoparasitic nematodes of small body length (<600 μm) with variable stylet length (10–120 μm), widely dispersed in different natural environments and crops, and distributed worldwide [2,3,4].

The taxonomic consideration for several genera of pin nematodes *sensu lato* historically included in this group comprise *Gracilacus*, *Paratylenchoide*s, *Gracilpaurus*, *Cacopaurus*, has been recently discussed by Singh et al. [3] concluding that all these genera were confirmed as synonyms with *Paratylenchus* since no clear separations were detected under phylogenetic relationships of ribosomal and mitochondrial genes [3]. Stylet drives the feeding habit and many species have a long stylet (>40 μm), becoming swollen and feeding from deeper layers in the root cortex as sedentary ectoparasites. Two stylet pattern shapes can be found in this genus: (a) long and flexible stylet > 40 μm with conus representing about more than 70% of the total stylet (m ratio), and juveniles with well-developed stylet, initially included in the genus *Gracilacus* Raski [5]; (b) short and rigid stylet < 40 μm with conus about 50% of the total stylet, and juveniles without well-developed stylet, initially included in the genus *Paratylenchus.* Nevertheless, these differences were not sufficiently supported by molecular analyses to separate and maintain both genera [3,4,6]. The genus *Paratylenchus* is a wide diverse group with about 130 nominal species, from which about 54 of them are molecularly characterized [2,3,4,6,7,8]. Consequently, about half of the nominal species of this genus are not yet linked to molecular data, and there is a need for completing that information. The conserved morphology that characterizes *Paratylenchus* species led to the development of molecular methods using different fragments of nuclear ribosomal and mitochondrial DNA gene sequences to be used in DNA barcoding [3,4,6,7,8]. Use of molecular markers in species identification of pin nematodes over the last years has indicated that many widespread species actually comprise multiple genetically divergent and morphologically similar cryptic species [3,4,6,7,8]. An emblematic example of these species complexes comprises the *Paratylenchus straeleni*-species complex, which Singh et al. [3] distinguished among 4–9 putative species within this complex considering all the available ribosomal and mitochondrial sequences. In 1988, Castillo and Gomez Barcina [9] identified a population of *P. straeleni* (De Coninck, 1931) Oostenbrink, 1960 from a natural environment (Portuguese oak forest, *Quercus faginea* Lam.) at southern Spain based on morphological and morphometric traits. This raises the possibility that this population was potentially misidentified and included under the common and widely distributed species *P. straeleni*. Consequently, this is an excellent opportunity which prompted us to apply integrative taxonomical approaches to unravel the cryptic diversity of this species complex. This study allowed us to verify if this species identification was correct or to prove if close morphology and morphometry with original description comprise some genetic diversity with recent molecularly studied *P. straeleni* populations from Belgium, USA, and Turkey [3,6,10,11].

Thirty species of *Paratylenchus* have been reported in Spain from cultivated and natural ecosystems including *P. aonli* Misra and Edward, 1971 [12], *P. arculatus* Luc and de Guiran, 1962 [13,14], *P. baldaccii* Raski, 1975 [4,15], *P. caravaquenus* Clavero Camacho, Cantalapiedra-Navarrete, Archidona-Yuste, Castillo and Palomares-Rius, 2021 [4], *P. ciccaronei* Raski, 1975 [16,17,18], *P. enatus* (Raski, 1976) Siddiqi, 1986 [19], *P. enigmaticus* Munawar, Yevtushenko, Palomares-Rius and Castillo, 2021 [4], *P. goodeyi* Oostenbrink, 1953 [20], *P. hamatus* Thorne and Allen, 1950 [4], *P. holdemani* Raski, 1975 [4], *P. indalus* Clavero Camacho, Cantalapiedra-Navarrete, Archidona-Yuste, Castillo and Palomares-Rius, 2021 [4], *P. israelensis* (Raski, 1973) Siddiqi, 1986 [4], *P. macrodorus* Brzeski, 1963 [18], *P. microdorus* Andrássy, 1959 [15,16,17,18], *P. minusculus* Tarjan, 1960 [21], *P. mirus* (Raski, 1962) Siddiqi and Goodey, 1964 [12], *P. nanus* Cobb, 1923 [18,22], *P. pedrami* Clavero-Camacho, Cantalapiedra-Navarrete, Archidona-Yuste, Castillo and Palomares-Rius, 2021 [4], *P. peraticus* (Raski, 1962) Siddiqi and Goodey, 1964 [20], *P. projectus* Jenkins, 1956 [19,23], *P. sheri* (Raski, 1973) Siddiqi, 1986 [16,17,18,22], *P. similis* Khan, Prasad and Mathur, 1967 [18,22], *P. steineri* Golden, 1961 [18,20], *P. straeleni* (De Coninck, 1931) Oostenbrink, 1960 [9], *P. tateae* Wu and Townshend (1973) [4], *P. tenuicaudatus* Wu, 1961 [12], *P. teres* (Raski, 1976) Siddiqi, 1986 [24], *P. vandenbrandei* de Grisse, 1962 [16,17], *P. veruculatus* Wu, 1962 [4], and *P. zurgenerus* Clavero-Camacho, Cantalapiedra-Navarrete, Archidona-Yuste, Castillo and Palomares-Rius, 2021 [4]. However, for the majority of these studies, except that of Clavero-Camacho et al. [4], no molecular analyses were carried out for their identification, and the cryptic biodiversity of these nematodes could be underexplored, including some species identifications for Spanish populations performed by our group some years ago. For this reason, the identification and reliable estimation of pin nematode diversity in Spain is needed. This paper is the second in a series deciphering the cryptic diversity of pin nematodes in Spain using integrative taxonomical approaches, with the final aim to disentangle the real biodiversity of these nematodes in cultivated and natural environments in Spain. The first one dealt with pin nematodes associated with cultivated *Prunus* spp. in Spain, including almond, apricot, cherry, nectarine and peach [4]. This study tries to understand the biodiversity of *Paratylenchus* spp. in some almond samples and additional new natural environments as well as re-analyzing some previous studies carried out by our laboratory 30 years ago based on morphology and morphometry only [9,17,20], but now using more accurate and precise integrative taxonomical approaches.

In the genus *Paratylenchus*, species display a particular resting-stage which accumulates in soil under adverse environmental conditions (*viz*. drought conditions) [4]. This state is non-feeding, molting to adults after stimulation by host-plant roots, and may provide some useful data for species identification [25,26]. Usually the resting stage is fourth-stage juvenile (J4), but third-stage (J3) appears in other species, recognized by granular body contents and presence/absence of stylet [2,26]. In *P. straeleni*, all juveniles had a well-developed stylet and pharynx, while the body of J4 contained numerous dark granules and this is considered the resting stage [26]. However, in close-related species such as *P. steineri*, stylet and pharynx are well-developed in second- and third-stage juveniles (J2 and J3), but J4 had no stylet and pharynx is much reduced. Morphological changes in stylet morphology in juveniles of some *Paratylenchus* species need to be studied with regard to adult state. In this research we study the stylet morphology of quiescent juvenile stages (J4) based on an integrative taxonomical approach [4].

The main objectives of this study were to (i) conduct identification with morphological and morphometrical approaches of some *Paratylenchus* species collected in several nematode surveys on almond and natural environments in Spain; (ii) provide molecular characterization of several species using ribosomal (D2-D3 expansion segments of 28S rRNA, Internal Transcribed Spacer region (ITS) rRNA) and the mitochondrial region cytochrome c oxidase subunit 1 (COI); (iii) study phylogenetic relationships within *Paratylenchus* spp. using the obtained molecular markers.

## 2. Results

Eighteen species were identified from 24 isolates of *Paratylenchus* spp. from 15 soil samples in nine municipalities in Spain (Table 1). In these populations, females, males (when available) and juveniles were morphologically and morphometrically studied in detail and molecular markers for their identification were provided (Table 1). From these, one isolate was considered a new undescribed species and 17 were already known described species (Table 1). The new species include an isolate from the *P. straeleni*-complex and was described herein as *Paratylenchus parastraeleni* sp. nov. The already known species included *P. amundseni* Bernard, 1982, *P. aciculus* Brown, 1959, *P. baldaccii* (Oostenbrink, 1953) Raski, 1962, *P. enigmaticus* Munawar et al., 2021, *P. goodeyi* Oostenbrink, 1953, *P. holdemani* Raski, 1975, *P. macrodorus* Brzeski, 1963, *P. neoamblycephalus* Geraert, 1965, *P. pandatus* (Raski, 1976) Siddiqi, 1986, *P. pedrami* Clavero-Camacho et al., 2021, *P. recisus* Siddiqi, 1996, *P. sheri* (Raski, 1973) Siddiqi, 1986, *P. tateae* Wu and Townshend (1973), *P. variabilis* Raski, 1975, *P. veruculatus* Wu, 1962, *P. verus* (Brzeski, 1995) Brzeski, 1998, and *P. vitecus* (Pramodini et al., 2006) Ghaderi et al., 2014. Eight of these species need to be considered as first reports for Spain in this research (*viz*. *P. amundseni*, *P. aciculus*, *P. neoamblycephalus*, *P. pandatus*, *P. recisus, P. variabilis, P. verus* and *P. vitecus*) and measurements from females, males (if available) and juveniles, as well as molecular markers were provided for their unequivocal identification.

### 2.1. Systematics

#### 2.1.1. Description of *Paratylenchus parastraeleni* sp. nov.

**Figure 1:**
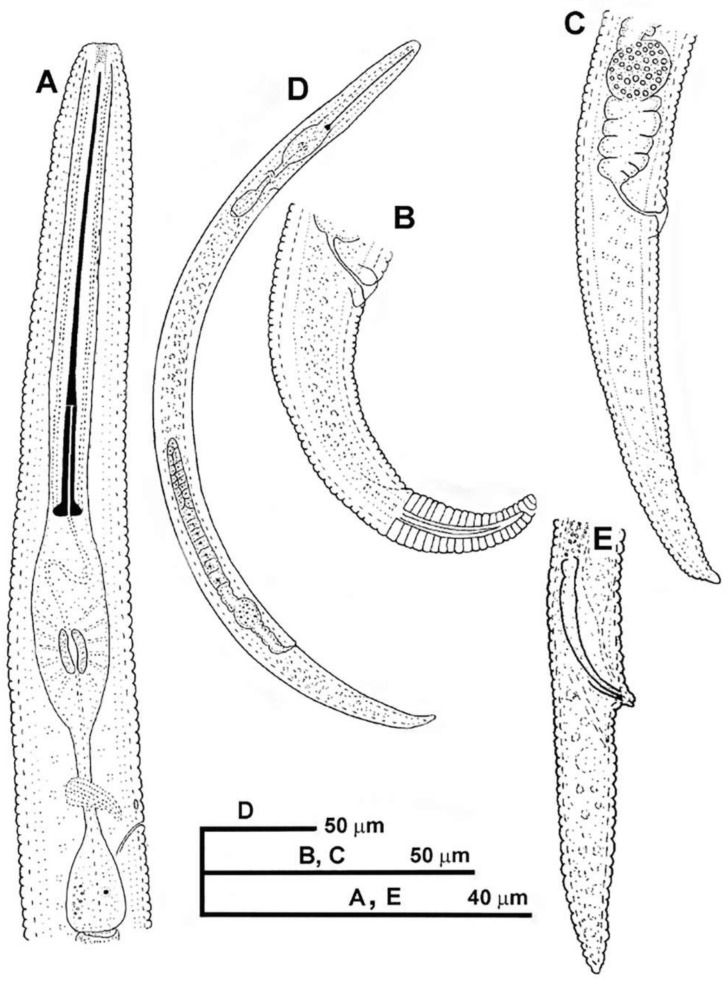
Line drawings of *Paratylenchus parastraeleni* sp. nov. (**A**): Female pharyngeal region; (**B**,**C**): Female posterior region; (**D**): Entire female; (**E**): Male posterior region.

**Figure 2:**
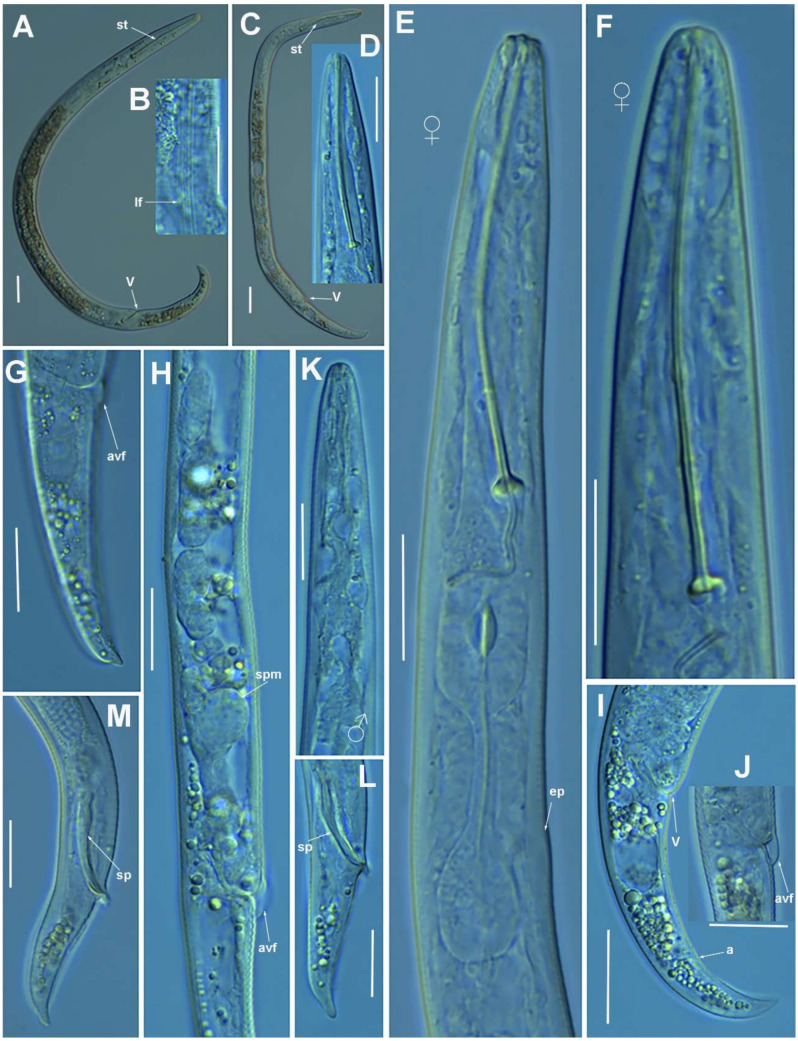
Light photomicrographs of *Paratylenchus parastraeleni* sp. nov. female and male. (**A**,**C**) Entire female with vulva arrowed; (**B**) detail of lateral fields; (**D**,**F**) detail of female stylet region; (**E**) female pharyngeal region; (**G**–**J**) female posterior region with vulva and anus (arrowed) and detail of vulva showing advulval flap (arrowed); (**K**) male pharyngeal region showing absence of stylet; (**L**,**M**) male posterior region showing spicules (arrowed). Scale bars (**A**–**M** = 20 μm). (Abbreviations: a = anus; avf = advulval flap; ep = excretory pore; lf = lateral field; sp = spicules; spm = spermatheca; V = vulva).

**Figure 3:**
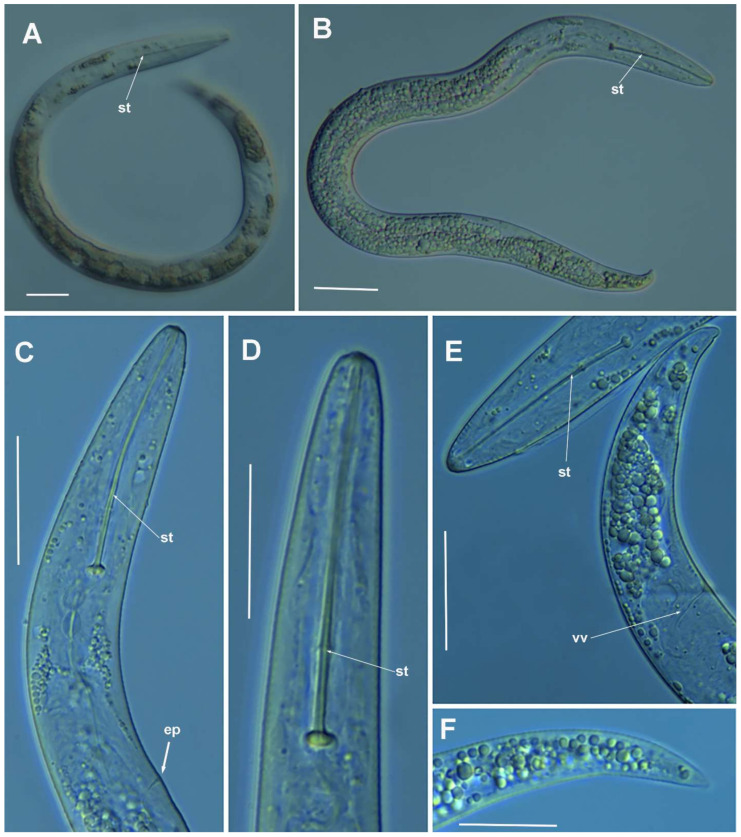
Light photomicrographs of *Paratylenchus parastraeleni* sp. nov. fourth-stage juveniles. (**A**,**B**) Entire fourth-stage juveniles showing stylet (arrowed); (**C**,**D**) fourth-stage juvenile pharyngeal region showing stylet; (**E**) fourth-stage juvenile anterior and posterior region showing stylet and initial vestigium of vagina (arrowed); (**F**) fourth-stage juvenile tail. Scale bars (**A**–**F** = 20 μm). (Abbreviations: ep = excretory pore; st = stylet; vv = vaginal vestigium).

(Figure 1, Figure 2 and Figure 3, Table 2) http://zoobank.org/urn:lsid:zoobank.org:act:61B40ACF-177F-4D92-A16F-0A3CAF78FD4A (accessed on 8 July 2021).

*Female*: body slender, ventrally arcuate to form an open, C-shaped body habitus when heat relaxed; cuticle finely annulated; lateral field equidistant with four distinct smooth lines. Lip region rounded, truncate, submedian lobes almost indistinct; with very slight sclerotization. Stylet flexible, 11.3–14.6% of body length. Conus of stylet 2.4–3.5 times longer than shaft, 73–80% of total stylet length. Stylet knobs small, 2.5–3.0 μm across, laterally directed. Procorpus cylindrical, about 50 μm long. Excretory pore situated at distal end of basal pharyngeal bulb. Hemizonid conspicuous, located two annuli anterior to excretory pore. Valvular apparatus in metacorpus 6.0–7.0 μm long, at 58–70% of pharynx length from anterior end. Basal pharyngeal bulb pyriform. Ovary outstretched, spermatheca almost spherical, 21 (19–28) μm wide, filled with rounded sperm 1.0–1.5 μm in diameter. Lateral vulval membranes, 5.5–6.0 μm long. Tail elongate-conoid gradually tapering to form a rounded terminus, 0.5–0.8 times as long as vulva–anus distance.

*Male*: Less common than females (ratio ca. 1:4). Male body is slender than female body, tapering towards both ends, posterior region ventrally arcuate when heat relaxed. Cuticle apparently smooth with fine annulations; labial region similar to that of female but narrower and slightly truncated, continuous with body, sclerotization in labial region weak; stylet lacking. Pharynx rudimentary and non-functional, procorpus, metacorpus, and basal bulb inconspicuous; excretory pore located 81.5 μm away from anterior end. Testis outstretched, with small spermatozoa; spicule slender, slightly curved towards end; gubernaculum curved; bursa absent. Tail elongate-conoid, tapering gradually to a finely pointed tip.

*Juveniles*: J4 similar in morphology to adult females (Figure 2 and Figure 3), bearing flexible stylet 45.8 (43.0–48.0) μm-long. Pharynx well developed, functional. Genital primordium underdeveloped, primordium of vagina discernible, anus indistinct, posterior region similar to female but slightly more rounded terminus.

##### Diagnosis and Relationships

The new species can be characterized by the presence of four lateral lines in lateral field, advulval flaps present, and a moderately long female stylet of 53.5 (52.0–56.0) µm. Lip region rounded, truncate, submedian lobes almost indistinct; with very slight sclerotization, continuous with the rest of the body. Spermatheca spherical. Tail elongate-conoid gradually tapering to form a rounded terminus. According to species grouping by Ghaderi et al. [2] belongs to group 10 characterized by stylet length more than 40 µm, four lateral lines and advulval flaps present.

Morphologically and morphometrically, the new species is very close to *P. straeleni*, and can be also similar to *P. goodeyi* and *P. ivorensis* Luc and de Guiran, 1962. In fact, the description of the Spanish population agrees well with original description by De Coninck [27], and other populations from The Netherlands, Poland, Italy, Czech Republic, Iran, USA, Turkey and Belgium [3,6,10,26,28,29,30], and no major differences in morphology or morphometry can be detected. Consequently, based on the molecular markers, this is an extraordinary example of cryptic species within the *P. straeleni*-complex species, and this can help to clarify the identity of other populations with similar morphology and morphometry. From *P. goodeyi* can be differentiated by lip region shape (conoid-rounded to truncate vs. conoid) [2], and from *P. ivorensis* in a posterior position of vulva (80.2–83.5 vs. 73–77).

##### Molecular Characterization

Seven D2-D3 of 28S rRNA (MZ265064-MZ265070), four ITS (MZ265004-MZ265007) and four COI gene sequences (MZ262208-MZ262211) were generated for this new species without intraspecific sequence variations, except for the ITS where only one variable position was detected. The closest species to *P. parastraeleni* sp. nov. was *P. straeleni*, being 95% similar for the D2-D3 region (MZ265064-MZ265070) (differing from 32 to 38 nucleotides and no indels) to several accessions deposited in GenBank. For the COI gene sequences (MZ262208-MZ262211), the similarity values were 93 and 94% (differing from 21 to 26 nucleotides and no indels) from *P. straeleni* sequences deposited in GenBank; finally, the similarity for the ITS region was 86–88% (differing from 89 to 111 nucleotides and 35 to 43 indels) from *P. straeleni* sequences deposited in GenBank. All molecular markers studied clearly separate both species. Due to the presence of more than one species in the same soil sample, J4 individual identification for morphological-morphometrical analysis was based on a molecular barcoding using the 28S rRNA markers, and nematodes with identical sequences as adults were considered as the same species, in this case, *P. parastraeleni* sp. nov.

##### Type Habitat and Locality

*Paratylenchus parastraeleni* sp. nov. was found in the rhizosphere of a *Quercus faginea* Lam., forest (coordinates 37°58′33.0″ N 2°54′18.8″ W); the municipal district of Arroyo Frío, Jaén province, Spain.

##### Etymology

The species epithet, *parastraeleni*, refers to Gr. prep. para, alongside of and resembling, because of its close resemblance to *Paratylenchus straeleni*.

##### Type Material

Holotype female, 17 paratypes females, 5 fourth-stage juveniles and 4 male paratypes (slide numbers CAZ_05-01 to CAZ_05-12) were deposited in the Nematode Collection of the Institute for Sustainable Agriculture, CSIC, Córdoba, Spain, and four females deposited at the USDA Nematode Collection (slides T-7511p and T-7512p).

#### 2.1.2. Remarks of *Paratylenchus aciculus* Brown, 1959

**Figure 4:**
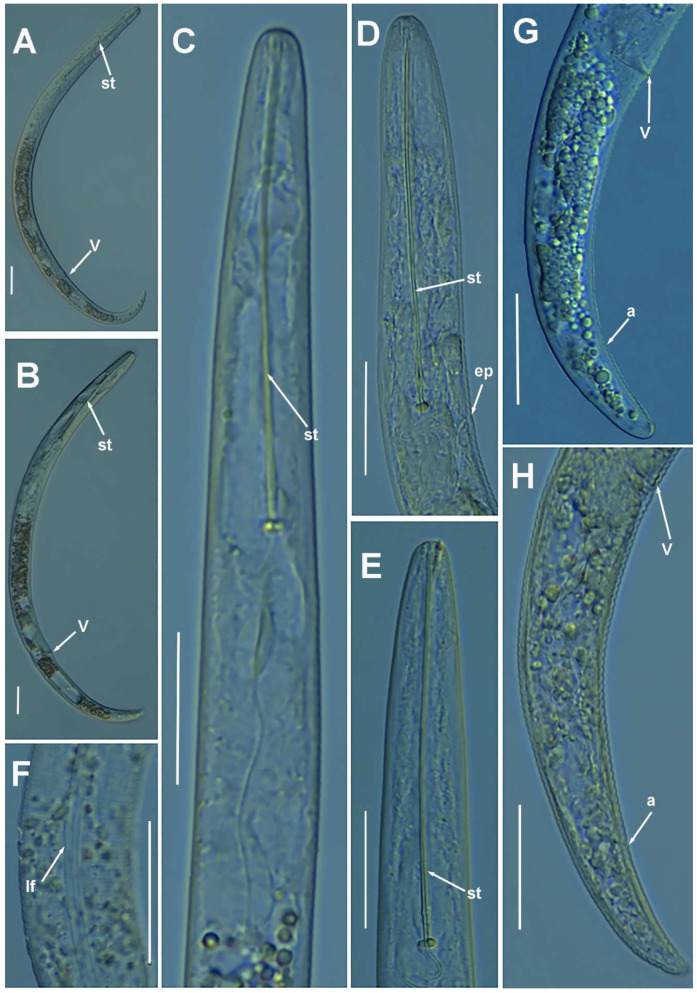
Light photomicrographs of *Paratylenchus aciculus* Brown, 1959. (**A**,**B**) Entire female with vulva arrowed; (**C**) female pharyngeal region; (**D**,**E**) female lip region; (**F**) detail of lateral field; (**G**,**H**) female posterior region with vulva and anus (arrowed). Scale bars (**A**–**H** = 20 μm). (Abbreviations: a = anus; ep = excretory pore; lf = lateral field; st = stylet; V = vulva).

(Figure 4, Table 3).

According to species grouping by Ghaderi et al. [2] this species belongs to group 9 characterized by stylet length more than 40 µm, three lateral lines and advulval flap absent. The Spanish population from Coto Ríos, Jaén province, was characterized by long flexible stylet 67.5–75.0 µm, lip region rounded and continuous with body contour, female tail subacute to finely rounded, and spermatheca ellipsoid and filled with sperm, which indicates that males are required for reproduction but their numbers are lower than females. J4 not found. Morphometrics of the Spanish population agree well with original description as well as other populations with small differences in stylet length (67.5–75.0 μm vs. 61.0–69.0 μm), which may be due to geographical intraspecific variability [2]. This species was described from Canada and has been reported in USA, several European countries, including the recent integrative identification from Belgium [3], and this study comprises the first report from Spain. Although ribosomal markers (D2–D3 and ITS) between the Spanish population of *P. aciculus* and the Belgian population of *P. aculentus* are quite similar (see below), these species can be separated by COI (see below), and by clear differences in stylet length (67.5–75.0 μm vs. 52.4–61.2 μm), advulval flap (absent vs. small advulval flap present), and spermatheca shape (ellipsoid vs. rounded) [3].

##### Molecular Characterization

Five D2-D3 of 28S rRNA (MZ265071-MZ265075), four ITS sequences (MZ265008-MZ265011), and three COI sequences (MZ262212-MZ262214) were obtained for this species. In both ribosomal genes, no intraspecific variability was detected, however, one variable position was found between the three COI sequences included in this study (MZ262212-MZ262214). Ribosomal genes (MZ265071-MZ265075, MZ265008-MZ265011) showed a high similarity with *P. aculentus*, being 99% (2 out of 698 bp difference) and 98% (11–12 out of 742 bp difference) similar for the D2-D3 (MW413626- MW413628) and ITS region (MW413588-MW413589), respectively. However, the separation of both species is possible using the COI gene (MZ262212-MZ262214), since for this marker the similarity found was 89% (differing by 40–41 nucleotides and no indels) with the accessions belonging to *P. aculentus* (MW421639-MW421641).

#### 2.1.3. Remarks of *Paratylenchus amundseni* Bernard, 1982

**Figure 5:**
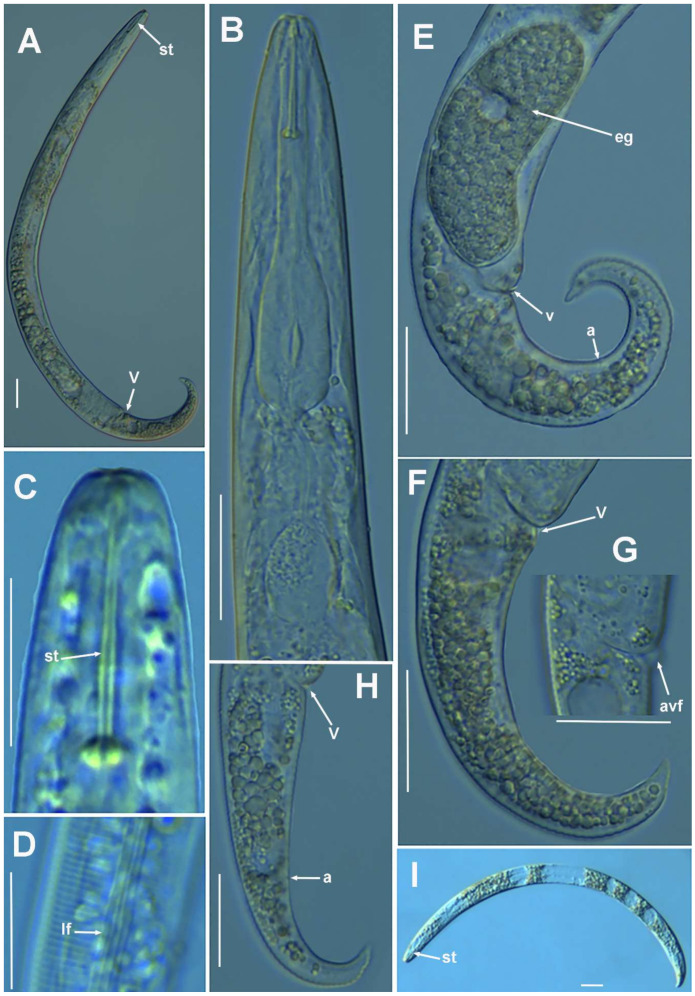
Light photomicrographs of *Paratylenchus amundseni* Bernard, 1982. (**A**) Entire female with vulva arrowed; (**B**) female pharyngeal region; (**C**) female lip region; (**D**) detail of lateral field; (**E**–**H**) female posterior region with vulva, anus, and advulval flap (arrowed); (**I**) entire fourth-stage juvenile with stylet (arrowed). Scale bars (**A**–**I** = 20 μm). (Abbreviations: a = anus; avf = advulval flap; eg = egg; lf = lateral field; st = stylet; V = vulva).

(Figure 5, Table 4).

According to species grouping by Ghaderi et al. [2] this species belongs to group 3 characterized by stylet length less than 40 µm, four lateral lines and advulval flaps present. The Spanish population from La Iruela, Jaén province, was characterized by a conoid-truncate lip region with submedian lobes indistinct, a female tail finely rounded to acute, and a rounded spermatheca filled with sperm, which indicates that males are required for reproduction but their numbers are lower than females. J4 bearing a delicate stylet. Some morphometric differences with original description include slightly larger body length (335–450 µm vs. 320–370 µm), slightly shorter stylet length (16.0–18.0 µm vs. 17.0–19.0 µm), and slightly posterior position of vulva (78.6–82.8 vs. 76.0–80.0), which may be considered as intraspecific variability. This species is very close morphologically and morphometrically to *P. tateae*, from which they can be separated by lip region (conoid-truncate and submedian lobes indistinct vs. conoid narrow, with anterior end flattened and protuberant submedian lips) (Figure 5), as well as by molecular markers (see below). This species has only been reported from original description in the rhizosphere of grasses (*Leymus mollis* (Trin.) Pilg.) at Adak Island, Alaska (USA) [32], and this consists of the first report from Spain and the second written record.

##### Molecular Characterization

Three D2-D3 of 28S rRNA (MZ265076-MZ265078), three ITS (MZ265012-MZ265014), and five COI gene sequences (MZ262215-MZ262219) were generated herein for this species, including J4 and female adult sequences. All sequences showed no intraspecific variation. *Paratylenchus amundseni* was molecularly closely related with *P. tateae*, showing similarity values of 98% (differing from 11 to 14 nucleotides and no indels) for D2-D3 region. However, for the ITS region, the similarity value was 95% (differing by 31 to 43 nucleotides and 7 to 11 indels) with *P. tateae* accessions (MW282766-MW282771) from Spain and Canada [8]. Finally, the similarity found for COI gene sequences was 90% (differing by 34–36 nucleotides) with the COI accessions of *P. tateae* (MZ262262-MZ262264) from Spain, newly obtained in the present study.

#### 2.1.4. Remarks on *Paratylenchus baldaccii* (Oostenbrink, 1953) Raski, 1962, *Paratylenchus enigmaticus* Munawar, Yevtushenko, Palomares-Rius and Castillo, 2021, *Paratylenchus holdemani* Raski, 1975, *Paratylenchus neoamblycephalus* Geraert, 1965, *Paratylenchus pedrami* Clavero-Camacho, Cantalapiedra-Navarrete, Archidona-Yuste, Castillo and Palomares-Rius, 2021, and *Paratylenchus veruculatus* Wu, 1962

(Table 5).

*Paratylenchus baldaccii*, *P. enigmaticus*, *P. holdemani*, *P. neoamblycephalus*, *P. pedrami*, and *P. veruculatus* have been previously recorded within recent studies of pin nematodes in Spain [4,15], and morphological and morphometrical data of them were coincident with previous reports. Consequently, only some morphometric data or D2-D3 sequences had been reported here for these nematode samples. *Paratylenchus baldaccii* was identified in grasses at Arroyo Frío, Jaén province, in the same sample that we previously identified a population of *P. vandenbrandei* [17]. These data suggest that most probably the previous record of *P. vandenbrandei* [17] needs to be considered as *P. baldaccii*, as well as other reports [16,33], but additional studies need to be carried out to confirm these potential misidentifications on the basis of application of integrative taxonomy. *Paratylenchus baldaccii* has been reported in several localities at south and southeastern Spain, including Jaén, Granada and Murcia provinces [4,15,22,34]. *Paratylenchus enigmaticus* was detected in the rhizosphere of grasses at campus Alameda del Obispo, Córdoba; this report confirms a wider distribution than previously estimated, since it was detected only in the rhizosphere of cherry at Northeastern of Spain at La Almunia, Zaragoza province [4]. *Paratylenchus holdemani* has been recently reported in the rhizosphere of almond at Martos, Jaén province [4]. This new report under a natural environment (wild olive) at St. Maria de Trasierra, Córdoba province, also suggests that this species can be common in Andalucia (Southern part of the Iberian Peninsula). Finally, *P. neoamblycephalus* was confirmed by molecular and morphometrical data under a natural environment (Portuguese oak forest). Unfortunately, only a mature female was detected (Table 5), but morphometrics agree with original description [35] and recent data by Singh et al. [3]. Consequently, up to our knowledge, this is the first report of this species for Spain. Finally, the new findings of *P. pedrami* and *P. veruculatus* from natural environments (wild olive) at Córdoba province confirms also that these species are widely distributed in Spain [4].

##### Molecular Characterization

Several populations of species already molecularly characterized in previous works, such as *P. baldaccii*, *P. enigmaticus*, *P. holdemani*, *P. neoamblycephalus*, *P. pedrami*, and *P. veruculatus* have been sequenced herein. All sequences obtained for these species matched well with the accessions from the same species deposited in GenBank, showing similarity values from 99 to 100% [3,4].

#### 2.1.5. Remarks on *Paratylenchus goodeyi* Oostenbrink, 1953

**Figure 6:**
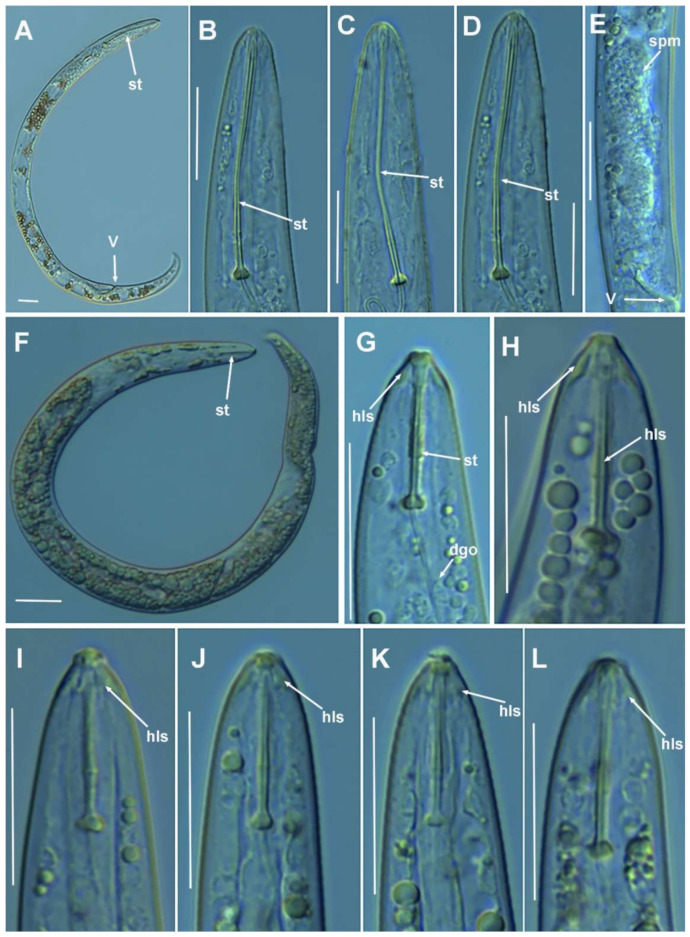
Light photomicrographs of *Paratylenchus goodeyi* Oostenbrink, 1953. (**A**) Entire female with vulva arrowed; (**B**–**D**) female lip region with stylet arrowed; (**E**) detail of vulval region showing spermatheca arrowed; (**F**) entire fourth-stage juvenile with short stylet arrowed; (**G**–**L**) fourth-stage juvenile lip regions showing labial sclerotization and short stylet (arrowed). Scale bars (**A**–**L** = 20 μm). (Abbreviations: dgo = pharyngeal dorsal gland orifice; hls = heavy labial sclerotization; spm = spermatheca; st = stylet; V = vulva).

(Figure 6, Table 6).

This species has been detected in several samples of almond and natural environment (wild olive) in several localities of Córdoba and Jaén provinces (Table 1). Morphology and morphometrics of adult females are coincident with the original description and recent studies [3,4]. However, in none of the previous studies on this species J4 were studied under an integrative taxonomic point of view. In all of our populations, irrespective of cultivated almond fields or natural environments, all the J4 of this species were characterized by bearing a short rigid and straight stylet (15.0–18.5 μm), lip region-truncate with labial framework sclerotization strong; with numerous dark granules into the body (Figure 6, Table 6), and considered the resting-stage [26]. In the original description of *P. goodeyi* it is mentioned that “J3 and J4 from soil samples, which probably belonged to this species, on account of the typical shape of the lip region, all had a short spear below 20 μm” [36]. However, this is the first report documenting, by morphometric and molecular markers (see below), a clear stylet and lip region metamorphosis between J4 and adult female, from short rigid stylet and conoid-truncate lip region with strong labial sclerotization moving to a long and slender flexible stylet and a conoid-rounded lip region without labial sclerotization (Figure 6). These data suggest, that apart from the reserve dark granules for resting during adverse environmental conditions (such as the hard drought during the summer season in Mediterranean climates), J4 of *P. goodeyi* is ready for feeding on susceptible roots during the beginning of the next season. Except for the stylet and lip region, J4 showed similar morphology to adult females with a posterior body rounded terminus. The present reports extend the geographical distribution of this species in Spain which has been already reported in several provinces including Navarra [12], Jaén [20,21], Barcelona [19], and Córdoba [4].

##### Molecular Characterization

Twenty-two D2-D3 sequences of 28S rRNA (MZ265084-MZ265105), 14 ITS (MZ265020-MZ265033), and 12 COI gene sequences (MZ262227-MZ262238) of *P. goodeyi* were generated in this study, with an intraspecific sequence variation from 0 to 9 nucleotides for D2-D3 of 28S rRNA (MZ265084-MZ265105), 0 to 17 nucleotides for ITS region (MZ265020-MZ265033), and finally, 0 to 29 nucleotides for COI gene (MZ262227-MZ262238). Some intraspecific sequence variations were detected when comparing with the accessions of *P. goodeyi* deposited in GenBank, showing similarity values of 99% for the D2-D3 of 28S rRNA, from 96 to 99% for the ITS region and finally, from 96 to 98% for the COI gene [3,4]. Some accessions from the different populations, belonging to J4, and all of them, matched well, from 99 to 100% similarity, with the sequences obtained for adult females of the same population.

#### 2.1.6. Remarks on *Paratylenchus macrodorus* Brzeski, 1963

**Figure 7:**
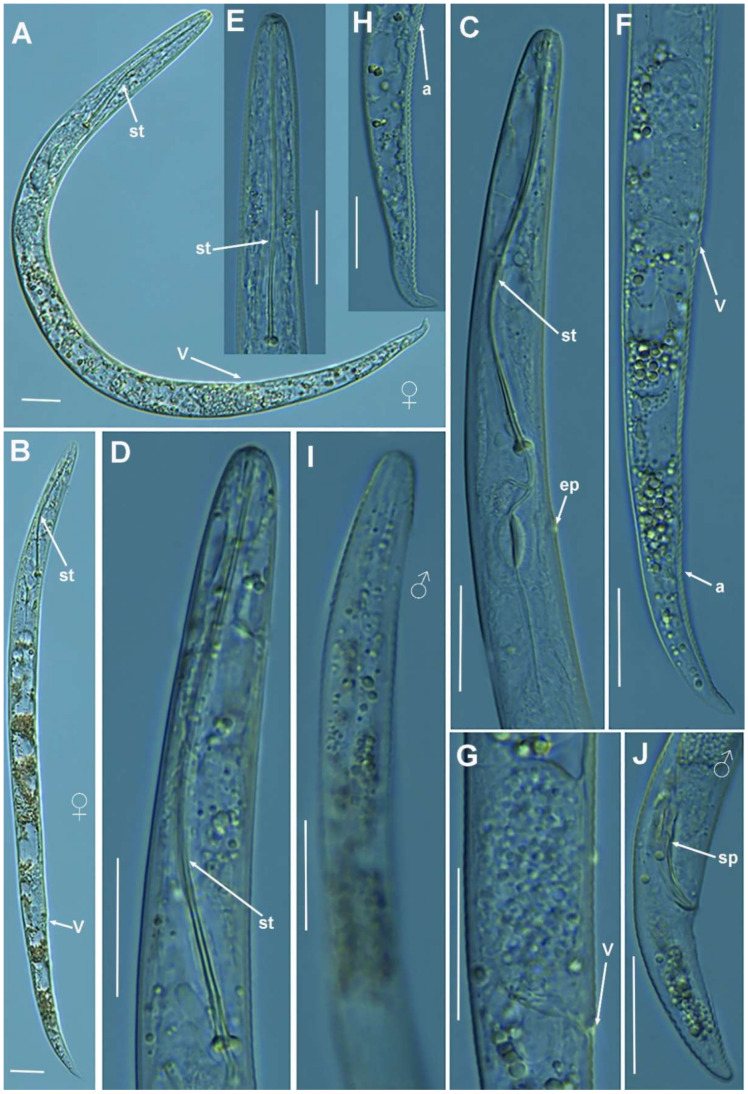
Light photomicrographs of *Paratylenchus* *macrodorus* Brzeski, 1963. (**A**,**B**) Entire female with stylet and vulva arrowed; (**C**) female pharyngeal region; (**D**,**E**) female lip region; (**F**) female posterior region with vulva and anus (arrowed); (**G**) detail of vulva (arrowed); (**H**) female tail region with anus arrowed; (**I**) male pharyngeal region showing absence of stylet; (**J**) male posterior region showing spicules (arrowed). Scale bars (**A**–**J** = 20 μm). (Abbreviations: a = anus; ep = excretory pore; sp = spicules; st = stylet; V = vulva).

(Figure 7, Table 7).

According to species grouping by Ghaderi et al. [2] this species belongs to group 11 characterized by stylet length more than 40 µm, four lateral lines and advulval flaps absent. The Spanish population from Santa Mª de Trasierra, Córdoba province, was characterized by long flexible stylet 70.0–84.0 µm, lip region continuous with body contour, tapering slightly to a blunt anterior end, submedian lobes fairly distinct, female tail tapering gradually to finely rounded terminus. Males without stylet, and J4 similar to female, except for shorter stylet (both stages confirmed belonging to this species by molecular markers). Morphometrics of the Spanish population agree well with original description as well as other populations with small differences in stylet length (70.0–84.0 μm vs. 75.0–92.0 μm), which may be due to geographical intraspecific variability [2]. Molecularly *P. macrodorus* is close to *P. pandatus* and *P. wuae* (using D2-D3 region of 28S rRNA) from which can be morphological and morphometrically separated by submedian lobes (fairly distinct vs. clearly distinct, pronounced submedian lobes, respectively), body length (317–410 vs. 290–339, 300–360 µm, respectively), c and c’ ratios (7.4–11.1 vs. 9.2–16.6, 10.5–11.3, and 3.5–4.9 vs. 2.2–3.0, 3.4–3.8, respectively), and J4 stylet (present vs. absent, absent, respectively) [2,37,38]. This species was described from vegetables from Poland [39] and has been reported from the Netherlands, Germany and Belgium [34], and New Caledonia [40]. This is the second report from Spain, the first being from natural environments in Almeria province [18].

##### Molecular Characterization

Six D2-D3 sequences of 28S rRNA (MZ265108-MZ265113), five ITS (MZ265034-MZ265038), and six COI gene sequences (MZ262239-MZ262244) were generated for *P. macrodorus* without intraspecific sequence variations for ribosomal genes, and J4 and adult female sequences were identical, confirming the identity of these juvenile individuals as *P. macrodorus*. *Paratylenchus macrodorus* showed high molecular similarity with *P. pandatus* and *P. wuae*, being 99% similar for the D2-D3 of 28S rRNA (varying from 3 to 7 nucleotides and no indels). For the ITS region, similarity values found for *P. macrodorus* ranging from 96% (34 nucleotides and 13 indels) to 98% (13 nucleotides and 2 indels) to *P. wuae* (KM061783) and *P. pandatus* (MZ265041-MZ265042), respectively. Similarity values detected in the COI gene were lower than in the ribosomal genes, being 96% (14 nucleotides and no indels) to *P. wuae* and 94% (24 nucleotides and no indels) to *P. pandatus.* However, morphologically and morphometrically *P. macrodorus*, *P. pandatus* and *P. wuae* can be clearly separated (see above).

#### 2.1.7. Remarks on *Paratylenchus pandatus* (Raski, 1976) Siddiqi, 1986

**Figure 8:**
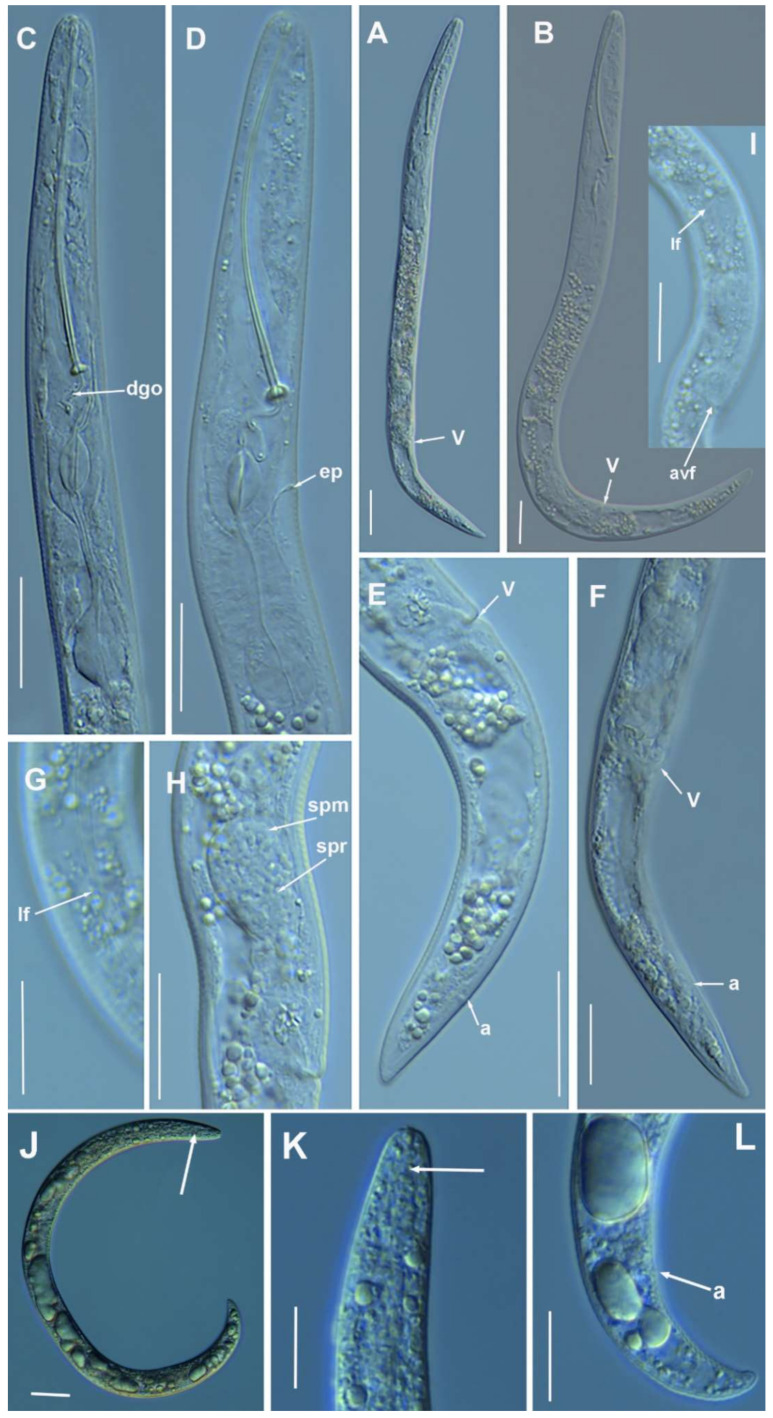
Light photomicrographs of *Paratylenchus* *pandatus* (Raski, 1976) Siddiqi, 1986. (**A**,**B**) entire females with vulva arrowed; (**C**,**D**) female pharyngeal region; (**E**,**F**) female posterior region showing vulva and anus (arrowed); (**G**) detail of lateral field (arrowed) at mid-body; (**H**) detail of spermatheca and sperm (arrowed); (**I**) detail of lateral field and advulval flap (arrowed); (**J**) entire fourth-stage juvenile, stylet absence arrowed; (**K**) fourth-stage juvenile lip region showing stylet absence (arrowed); (**L**) fourth-stage juvenile tail. Scale bars (**A**–**L** = 20 μm). (Abbreviations: a = anus; avf = advulval flap; dgo = pharyngeal dorsal gland orifice; ep = excretory pore; lf = laterl field; spm = spermatheca; spr = sperm; V = vulva).

(Figure 8, Table 8).

According to species grouping by Ghaderi et al. [2] this species belongs to group 10 characterized by stylet length more than 40 µm, four lateral lines and advulval flaps present. The Spanish population from Caravaca, Murcia province, was characterized by moderately long flexible stylet 57.0–68.5 µm, lip region rounded, continuous with body contour, with distinct submedian lobes, spermatheca elongate and filled with sperm, which indicates that males are required for reproduction but were not detected, female tail tapering gradually to rounded terminus. J4 was similar to female, except for absent stylet (stages confirmed belonging to this species by molecular markers). Morphometrics of the Spanish populations agree well with the original description, as well as Vietnam population with small differences in stylet length (57.0–68.5 μm vs. 63.0–70.0 μm), V ratio (74.5–77.7 vs. 70.0–76.0), and shape of tail terminus (finely rounded in Spanish and Vietnam populations while almost acute in original description), which may be due to geographical intraspecific variability [2]. This species was described from grapefruit in Nigeria [37] and has been reported from Vietnam [41] and Ethiopia [42], and this study comprises the first report from Spain. This species is closely related molecularly to *P. macrodorus*, but they have important morphological differences such as the presence vs. absence of advulval flaps and J4 without stylet vs. J4 with stylet.

##### Molecular Characterization

Two identical D2-D3 of 28S rRNA (MZ265116-MZ265117), two identical ITS sequences (MZ265041-MZ265042) and five identical COI gene sequences (MZ262247-MZ262251) were obtained from *P. pandatus* in the present study. Sequences obtained from J4 and females for all genes were identical, confirming that are the same species. *Paratylenchus pandatus* showed high molecular similarity with *P. macrodorus* and *P. wuae*, being 99% similar for the D2-D3 of 28S rRNA (varying from 7 to 8 nucleotides and no indels). For the ITS region, the similarity values were from 97% to 98% (differing by 13–15 nucleotides and from 2 to 6 indels) with *P. macrodorus* and *P. wuae,* respectively. Similarity values detected in the COI gene were lower than in the ribosomal genes, being 93% (23 nucleotides and no indels) to *P. wuae* and 94% (24 nucleotides and no indels) to *P. macrodorus.*

#### 2.1.8. Remarks on *Paratylenchus recisus* Siddiqi, 1996

**Figure 9:**
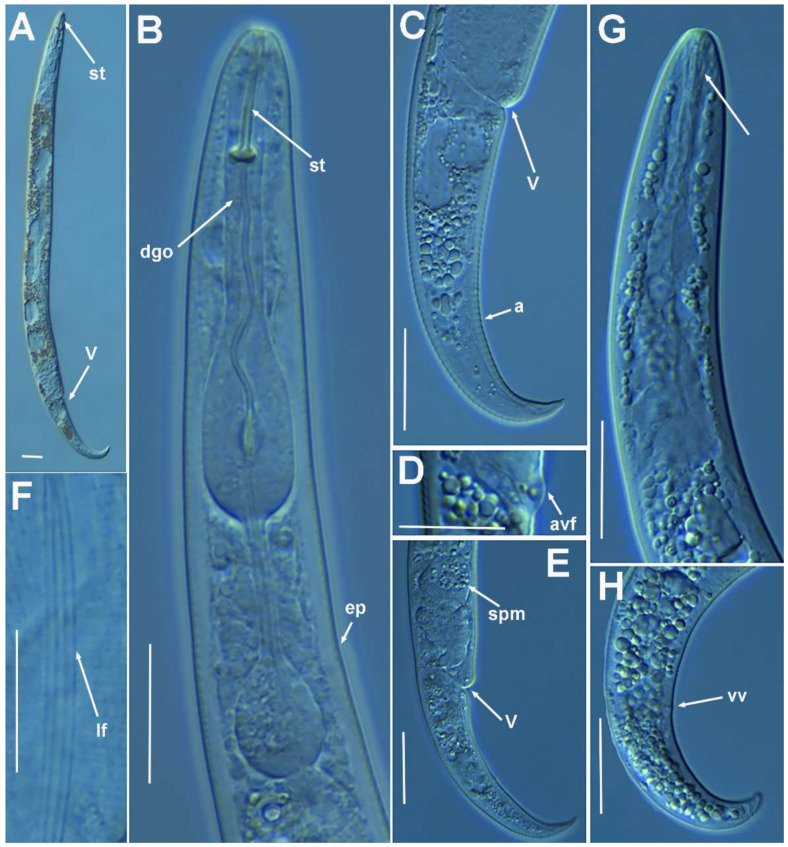
Light photomicrographs of *Paratylenchus* *recisus* Wu, 1974. (**A**) Entire female with stylet and vulva arrowed; (**B**) female pharyngeal region; (**C**–**E**) female posterior region with vulva, advulval flap, spermatheca and anus (arrowed); (**F**) detail of lateral field at mid-body (arrowed); (**G**) fourth-stage juvenile pharyngeal region showing absence of stylet (arrowed) and undeveloped pharynx; (**H**) fourth-stage juvenile posterior region showing vaginal vestigium (arrowed). Scale bars (**A**–**H** = 20 μm). (Abbreviations: a = anus; avf = advulval flap; dgo = pharyngeal dorsal gland orifice; ep = excretory pore; lf = lateral field; spm = spermatheca; st = stylet; vv = vaginal vestigium; V = vulva).

(Figure 9, Table 9).

According to species grouping by Ghaderi et al. [2] this species belongs to group 3 characterized by stylet length less than 40 µm, four lateral lines and advulval flaps present. The Spanish population from Arroyo Frío, Jaén province, was characterized by a short stylet 14.5–16.0 µm with rounded basal knobs, lip region rounded to truncate, continuous with body contour, indistinct submedian lobes, spermatheca rounded and filled with sperm, which indicates that males are required for reproduction but were not detected, female tail ventrally arcuate, tapering gradually to rounded terminus. J4 was similar to female, except for absent stylet (stage confirmed belonging to this species by molecular markers). Morphometrics of the Spanish population agree well with original description from Colombia [43], with small differences in stylet length (14.5–16.0 µm vs. 15.0–17.0 μm), c’ ratio (2.8–3.5 vs. 2.7–3.3), vulva–anus distance (1.5–1.8 times tail length), and tail length (28.0–34.0 µm vs. 18.0–29.0 μm), which may be due to geographical intraspecific variability. This species was described from Llanos Oriental in Colombia [43] and this study comprises the first report from Spain. This species is morphologically close to *P. microdorus*, from which can be differentiated by vulva–anus distance with regard to tail length and tail terminus, and probably has been misidentified in some previous records with *P. microdorus*, therefore additional studies need to clarify the real biodiversity in the *P. microdorus*-species complex in Spain by applying integrative taxonomy.

##### Molecular Characterization

Two D2-D3 of 28S rRNA (MZ265119-MZ265120), one ITS (MZ265043), and one COI gene sequence (MZ262252) were generated herein without intraspecific sequence variations. The closest *Paratylenchus* sequences to *P. recisus* were those of *P. microdorus* with 97, 93 and 91% similarity for the D2-D3 of 28S rRNA, ITS region and COI gene (MW421666-MW421667), respectively.

#### 2.1.9. Remarks on *Paratylenchus sheri* (Raski, 1973) Siddiqi, 1986

**Figure 10:**
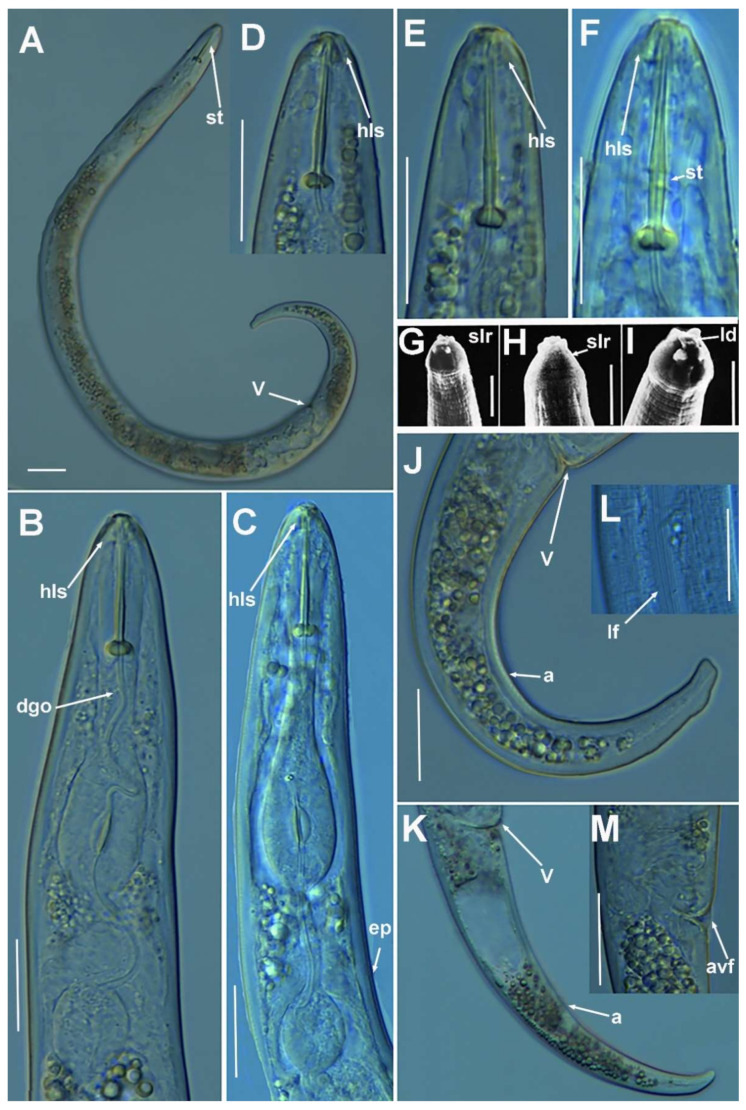
Light and SEM photomicrographs of *Paratylenchus* *sheri* (Raski, 1973) Siddiqi, 1986. (**A**) Entire female with stylet and vulva arrowed; (**B**,**C**) female pharyngeal region showing heavy lip sclerotization (arrowed); (**D**–**F**) female lip region showing heavy lip sclerotization (arrowed); (**G**–**I**) detail of lip region at SEM showing smooth lip region and labial disc (arrowed); (**J**,**K**) female posterior region with vulva and anus (arrowed); (**L**) detail of lateral field at mid body (arrowed); (**M**) detail of vulva showing advulval flap (arrowed). Scale bars (**A**–**F** = 20 μm; **G**–**I** = 5 μm; **J**–**M** = 20 μm). (Abbreviations: a = anus; avf = advulval flap; dgo = pharyngeal dorsal gland orifice; ep = excretory pore; hls = heavy lip sclerotization; ld = labial disc; slr = smooth lip region; st = stylet; V = vulva).

**Figure 11:**
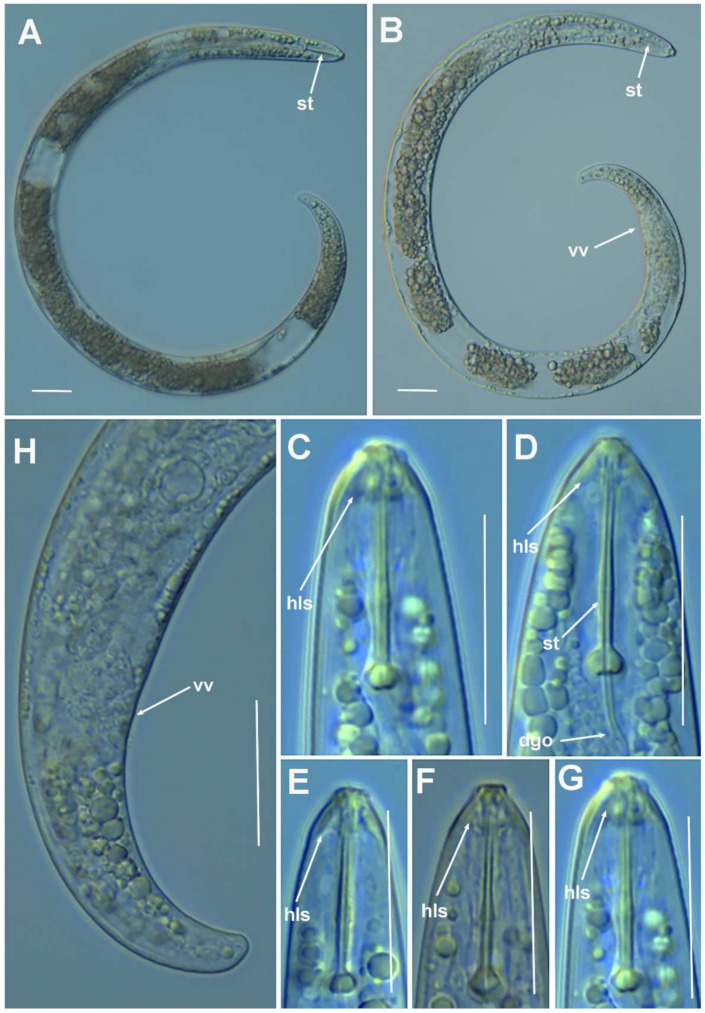
Light photomicrographs of *Paratylenchus* *sheri* (Raski, 1973) Siddiqi, 1986. (**A**,**B**) Entire fourth-stage juvenile with stylet and vaginal vestigium arrowed; (**C**–**G**) fourth-stage juvenile lip region showing heavy lip sclerotization (arrowed); (**H**) fourth-stage juvenile with vaginal vestigium arrowed. Scale bars (**A**–**H** = 20 μm). (Abbreviations: dgo = pharyngeal dorsal gland orifice; hls = heavy lip sclerotization; st = stylet; vv = vaginal vestigium).

(Figure 10 and Figure 11, Table 10).

According to species grouping by Ghaderi et al. [2] this species belongs to group 3 characterized by stylet length less than 40 µm, four lateral lines and advulval flaps present. The Spanish population from Arroyo Frío, Jaén province, was characterized by a conoid-truncate lip region, with an unstriated depression from body contour (4–5.5.0 µm wide) and strong sclerotization. Small projecting oral lips present. SEM face view (Figure 10) shows an unstriated, dorso-ventrally flattened lip region. Stylet robust, occupying 19 (15–23) annuli and 22.5–25.0 µm long. Stylet knobs weakly backwardly directed, 4.8 (4.0–5.5) µm across. Lateral field with four incisures with smooth margins (central two are very faint), 3.2 (2.5–4.0) µm wide. Orifice of dorsal pharyngeal gland 5.9 (4.5–6.5) µm from stylet base. Metacorpus with well-developed valvular apparatus 5.6 (4.5–7.5) µm long, its posterior margin situated at 70 (63–80) µm from anterior end. Excretory pore located near anterior end of basal bulb, immediately posterior to hemizonid. Cardia well developed, 2.5–3.5 µm wide. Distinct cuticular vulval flap, 6 (5.0–7.0) µm long. Large round spermatheca 13.5 (11.5–15.5) µm wide, filled with sperms 1–2 µm wide, which indicates that males are required for reproduction but their numbers are lower than females. Tail almost straight to slight ventrally curved, with rounded terminus, 0.8 (0.6–1.3) times vulva–anus distance or 3.8 (3.1–4.7) times anal body diameter. J4 with similar morphology to that of adult females, except sexual characters and shorter body length and stylet.

This species was described from Digne, France [44], and has been reported in Spain [17,18] and Italy [28]. This population was from the same locality as that reported by Gomez-Barcina et al. [17], which was confirmed by Prof. Raski [17]. The species was recently synonymized with *P. israelensis* by Ghaderi et al. [2] based on similar morphology, including strong labial sclerotization. However, the present results together with the recent integrative taxonomical diagnosis of *P. israelensis* [4] demonstrated that both species are closely related morphologically and molecularly (see below) and need to be considered as nominal valid species. This species has also been reported in Iran [45]; however, the single D2-D3 sequence provided for this Iranian population was 99.7% similar to *P. tateae* from Spain and Canada (see below) and needs to be revised by the authors.

##### Molecular Characterization

Six D2-D3 of 28S rRNA (MZ265121-MZ265126) with an intraspecific sequence variation of 0.5% (differing from 0 to 2 nucleotides), seven ITS (MZ265044-MZ265050) (99% similarity; nine nucleotides and no indels), and finally, nine COI gene sequences (MZ262253-MZ262261), with an intraspecific sequence variation of 5% (differing from 0 to 23 nucleotides), were generated. Two J4 from the Arroyo Frío population were sequenced, including D2–D3 of 28S (MZ265121-MZ265122), ITS region (MZ265044-MZ265045) and COI gene (MZ262253-MZ262254) being identical to the adult female sequences from this population. The D2-D3 of 28S rRNA sequences (MZ265121-MZ265126) showed high similarity with accession from *P. israelensis* (MW798301-MX798305) and *P. neoamblycephalus* (MW413660-MW413663) being 99% similar between them (differing from 2 to 7 nucleotides). For the ITS (MZ265044-MZ265050), the similarity detected was 98% (differing by 17–23 nucleotides and 6–8 indels) with *P. israelensis* (MW798343) and 95% (differing by 44–50 nucleotides and 20 indels) with *P. neoamblycephalus* (MW413607). Finally, for the COI gene sequences (MZ262253-MZ262261), *P. sheri* showed similarity values of 92–94% (differing from 21 to 29 nucleotides) with *P. israelensis* (MW797019-MW797020) and 89–91% (differing from 34 to 38 nucleotides) with *P. neoamblycephalus* (MW421677-MW421682). D2–D3 of 28S sequences from *P. sheri* obtained herein showed similarity values of 91% with the accession MN088374 of *P. sheri* from Iran, thus reinforcing the idea that this sequence belongs to *P. tateae* instead of *P. sheri,* as already suggested by Munawar et al. [8].

#### 2.1.10. Remarks on *Paratylenchus variabilis* Raski, 1975

**Figure 12:**
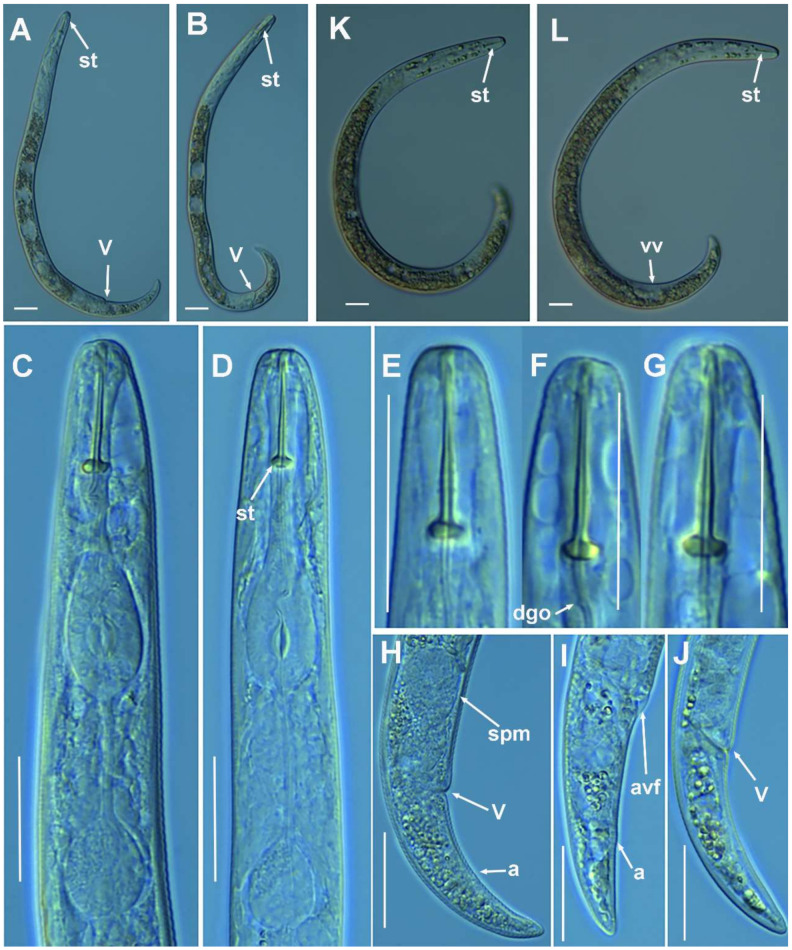
Light photomicrographs of *Paratylenchus* *variabilis* Raski, 1975. (**A**,**B**) Entire female with stylet and vulva arrowed; (**C**,**D**) female pharyngeal region; (**E**–**G**) female lip region; (**H**–**J**) female posterior region with vulva, spermatheca, advulval flap and anus (arrowed); (**K**–**L**) fourth-stage juvenile with stylet and vaginal vestigium arrowed. Scale bars (**A**–**L** = 20 μm). (Abbreviations: a = anus; avf = advulval flap; dgo = pharyngeal dorsal gland orifice; spm = spermatheca; st = stylet; vv = vaginal vestigium; V = vulva).

(Figure 12, Table 11).

According to species grouping by Ghaderi et al. [2] this species belongs to group 3 characterized by stylet length less than 40 µm, four lateral lines and advulval flaps present. The Spanish population from Córdoba, Córdoba province, was characterized by a rounded lip region with indistinct submedian lobes, continuous with the rest of the body, short stylet 14.0–16.0 µm long, spermatheca oval and filled with sperm, which indicates that males are required for reproduction but were not found, and female tail narrows gradually to a bluntly rounded terminus. J4 with similar morphology to that of adult females, except sexual characters and shorter body length and stylet. This species was described from California and Utah [46] and has been reported in Israel and Iran [30], and this study comprises the first report from Spain. This species is morphologically close to *P. microdorus*, from which can be differentiated by the shape of female tail terminus, and probably has been misidentified in previous records with *P. microdorus*, therefore, additional studies need to clarify the real biodiversity in the *P. microdorus*-species complex in Spain by applying integrative taxonomy. In addition, *P. variabilis* is morphologically and morphometrically almost indistinguishable from *P. zurgenerus* [4], from which it can be separated by molecular markers (see below), and both can be considered as cryptic species.

##### Molecular Characterization

Three D2-D3 of 28S rRNA (MZ265127-MZ265129), three ITS (MZ265051-MZ265053) and three COI gene sequences (MZ262265-MZ262267) were generated in this study from two adult females and one J4 specimen without intraspecific sequence variations. *Paratylenchus variabilis* was closely related with *P. nanus*, showing similarity values of 96% (differing by 31 nucleotides and 1 indel) for the D2-D3 region with several accessions of *P. nanus* (MW413657-MW413659, MW234449-MW234450). However, for the ITS region the similarity was lower, with values about 87% with accessions belonging to several *Paratylenchus* spp., such as, *P. veruculatus*, *P. goodeyi* and *P. nanus*. Finally, the closest species for the COI gene sequences was *P. goodeyi* (MW421648-MW421649), being 95% similar between them (19 nucleotides and no indels). Finally, *P. variabilis* can also be clearly separated molecularly from *P. zurgenerus* by D2-D3 and ITS, 88.9%, 78.3% similarity (differing in 79 bp, 166 and 20, 66 indels), respectively; low similarity was detected among COI sequences of both species.

#### 2.1.11. Remarks on *Paratylenchus verus* (Brzeski, 1995) Brzeski, 1998

**Figure 13:**
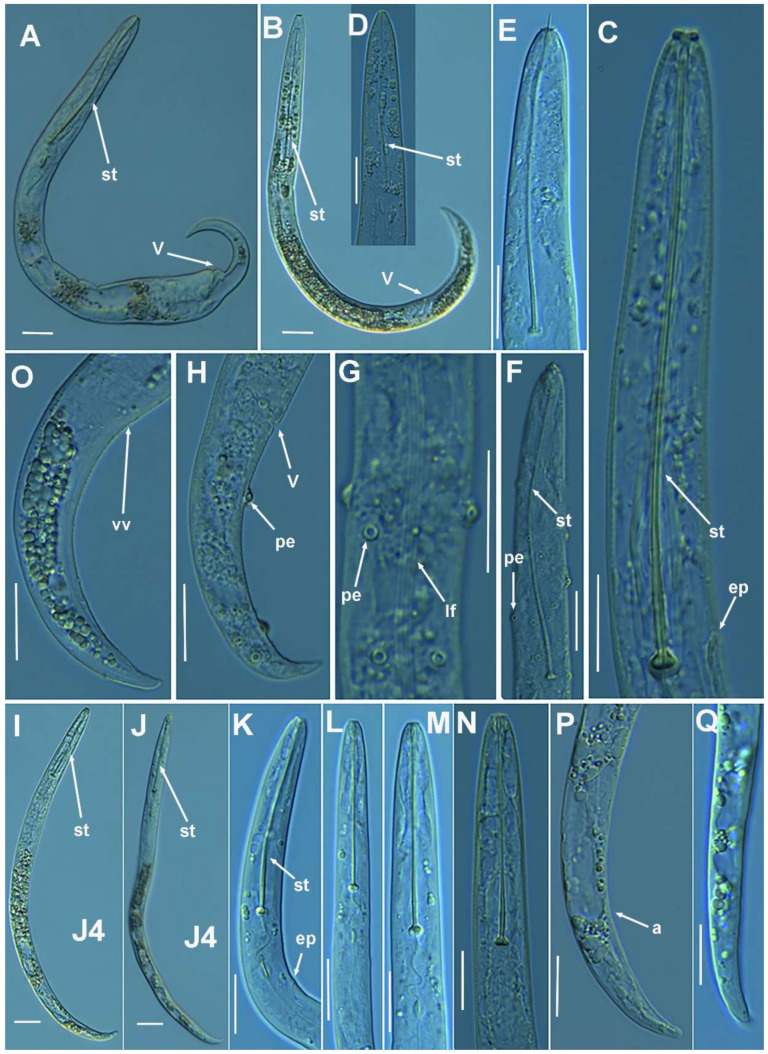
Light photomicrographs of *Paratylenchus* *verus* (Brzeski, 1995) Brzeski, 1998. (**A**,**B**) Entire female with stylet and vulva arrowed; (**C**–**F**) female lip region with stylet, excretory pore and *Pasteuria* endospore arrowed; (**G**) detail of lateral field at mid-body and *Pasteuria* endospore arrowed; (**H**) female posterior region with vulva and *Pasteuria* endospore arrowed; (**I**,**J**) entire fourth-stage juvenile with stylet arrowed; (**K**–**N**) fourth-stage juvenile lip region with stylet and excretory pore arrowed; (**O**) fourth-stage juvenile posterior region with vaginal vestigium arrowed; (**P**,**Q**) fourth-stage juvenile posterior region with anus arrowed. Scale bars (**A**–**Q** = 20 μm). (Abbreviations: a = anus; ep = excretory pore; lf = lateral field; pe = *Pasteuria* endospore; st = stylet; vv = vaginal vestigium; V = vulva).

(Figure 13, Table 12).

According to species grouping by Ghaderi et al. [2] this species belongs to group 10 characterized by stylet length more than 40 µm, four lateral lines and advulval flaps present. The Spanish population from Sta. Maria de Trasierra, Córdoba province, was characterized by a rounded lip region with distinct submedian lobes, continuous with the rest of the body, long flexible stylet 79.0–97.0 µm long, excretory pore opposite to median bulb, spermatheca oval and filled with sperm, which indicates that males are required for reproduction but were not found, and female tail narrows gradually to a rounded terminus. J4 with similar morphology to that of adult females, except sexual characters and shorter body length and stylet. This species was described from Texcoco, Mexico [28], and this study comprises the first report from Spain. Several females of the Spanish population had conspicuous infections of *Pasteuria* sp. on cuticle, especially on anterior and posterior ends (Figure 13).

##### Molecular Characterization

Four D2-D3 of 28S rRNA (MZ265130-MZ265133), five ITS (MZ265054-MZ265058) and four COI (MZ262268-MZ262271) gene sequences were generated for the first time from this species, including J4 and adult females, without intraspecific sequence variations, except for the ITS sequences with 98–100% similarity (differing from 2 to 11 nucleotides and 0 to 2 indels). The closest *Paratylenchus* spp. was *P. idalimus* being 96% similar (22 nucleotides and no indels) for the D2-D3 of 28S rRNA, 90% similar for the ITS region (differing by 69–75 nucleotides and from 21 to 23 indels) and, finally, 93% for COI sequences (MW411839) (differing by 26 nucleotides and no indels).

#### 2.1.12. Remarks on *Paratylenchus vitecus* (Pramodini et al., 2006) Ghaderi et al., 2014

**Figure 14:**
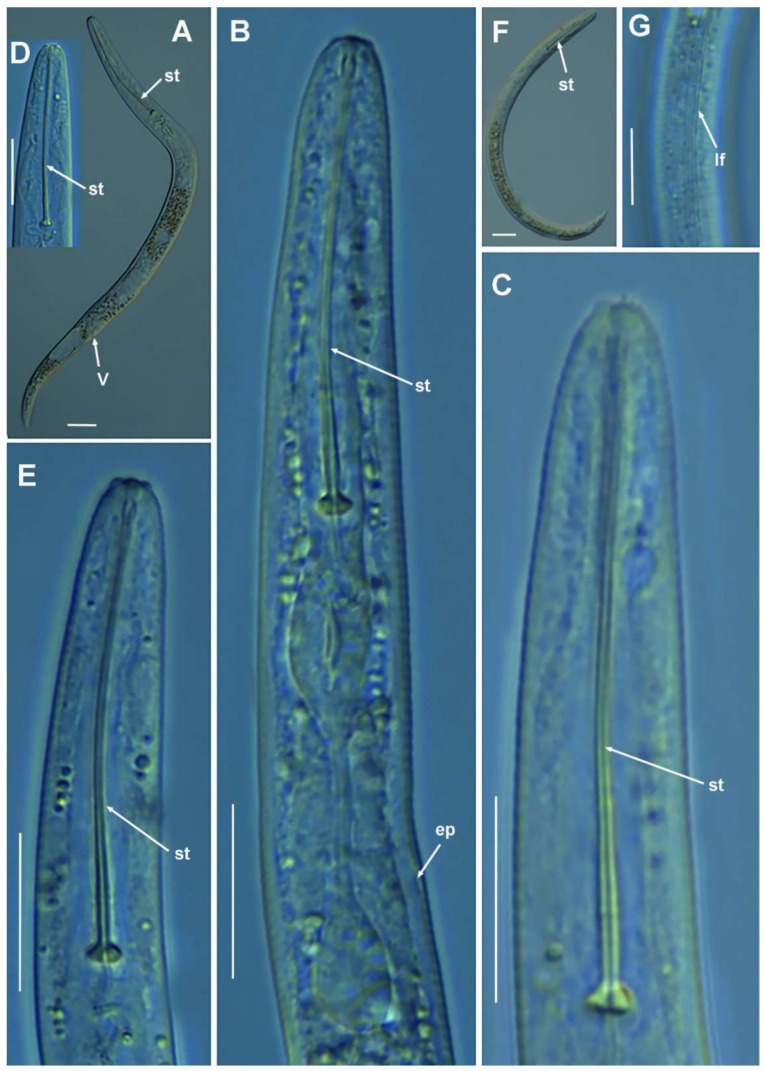
Light photomicrographs of *Paratylenchus* *vitecus* (Pramodini et al., 2006) Ghaderi et al., 2014. (**A**) Entire female with stylet and vulva arrowed; (**B**) female pharyngeal region with stylet and excretory pore arrowed; (**C**–**E**) female lip region; (**F**) entire fourth-stage juvenile with stylet arrowed; (**G**) detail of lateral field at mid-body (arrowed). Scale bars (**A**–**G** = 20 μm). (Abbreviations: ep = excretory pore; lf = lateral field; st = stylet; V = vulva).

(Figure 14, Table 13).

According to species grouping by Ghaderi et al. [2] this species belongs to group 11 characterized by stylet length more than 40 µm, four lateral lines in and advulval flaps absent. The Spanish population from Córdoba, Córdoba province, was characterized by a conoid-rounded lip region with distinct submedian lobes, continuous with the rest of the body, long flexible stylet 62.0–70.0 µm long, spermatheca elongate and filled with sperm, which indicates that males are required for reproduction but not found, and female tail finely rounded. J4 with similar morphology to that of adult females, except sexual characters and shorter body length and stylet. Morphometrics of the Spanish population agree well with original description with small differences in stylet length (62.0–70.0 μm vs. 42.0–65.0 μm), V ratio (68.1–75.4 vs. 72.0–77.0), which may be due to geographical intraspecific variability [2]. Molecularly, *P. vitecus* is close to *P. teres* (see below), however, it can be morphologically separated by clear differences in stylet length (42.0–65.0 μm, 62.0–70.0 μm vs. 69.0–83.0 μm, 67.0–96.0 μm) and c’ ratio (2.9, 2.7–3.5 vs. 4.2, 3.1–3.9). This species was described from Manipur, India [47], and this study comprises the first report from Spain.

##### Molecular Characterization

Six D2-D3 of 28S rRNA (MZ265136-MZ265141) and four ITS (MZ265059-MZ265062) with one and two variable positions, respectively, and three identical COI gene sequences (MZ262272-MZ262274) were generated for this species, including sequences from J4 and adult females. The closest *Paratylenchus* spp. was *P. teres* with 97% similarity for the D2-D3 of 28S rRNA (differing by 25 nucleotides) to MN088376. Unfortunately, no data for ITS or COI from *P. teres* are available in the GenBank.

### 2.2. Distribution of Paratylenchus spp. in Spain

**Figure 15:**
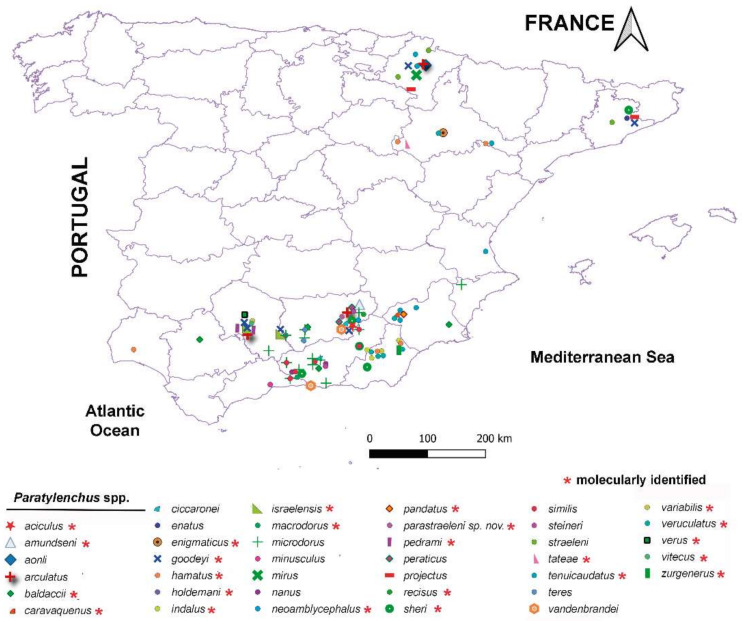
Spain map distribution of *Paratylenchus* species across all of the country. Species list with asterisk (*) indicated species identified by integrative taxonomy and including molecular analyses confirmation.

In the exhaustive review of the geographical distribution of Paratylenchus species in cultivated and natural environments in Spain, we detected that pin nematodes exhibited a wide distribution across an extensive variety of herbaceous and woody hosts, including 39 species (Figure 15). It should be noted that the highest diversity seems to be associated with southern Spain (Andalucia), with 35 out of 39 species in the country (Figure 15). Although the data suggest that the nematode survey efforts were higher in southern than in central and northern parts of the country, the biodiversity of Paratylenchus in Andalucia is really remarkable (Figure 15). In any case, the Paratylenchus species distribution observed herein revealed that this genus is adapted to a wide variety of host plants and heterogeneous environmental conditions (climatic, edaphic) from all over the country (ca. 1000 km across north–south, and ca. 600 km across east–west).

### 2.3. Phylogenetic Analyses of Paratylenchus spp.

**Figure 16:**
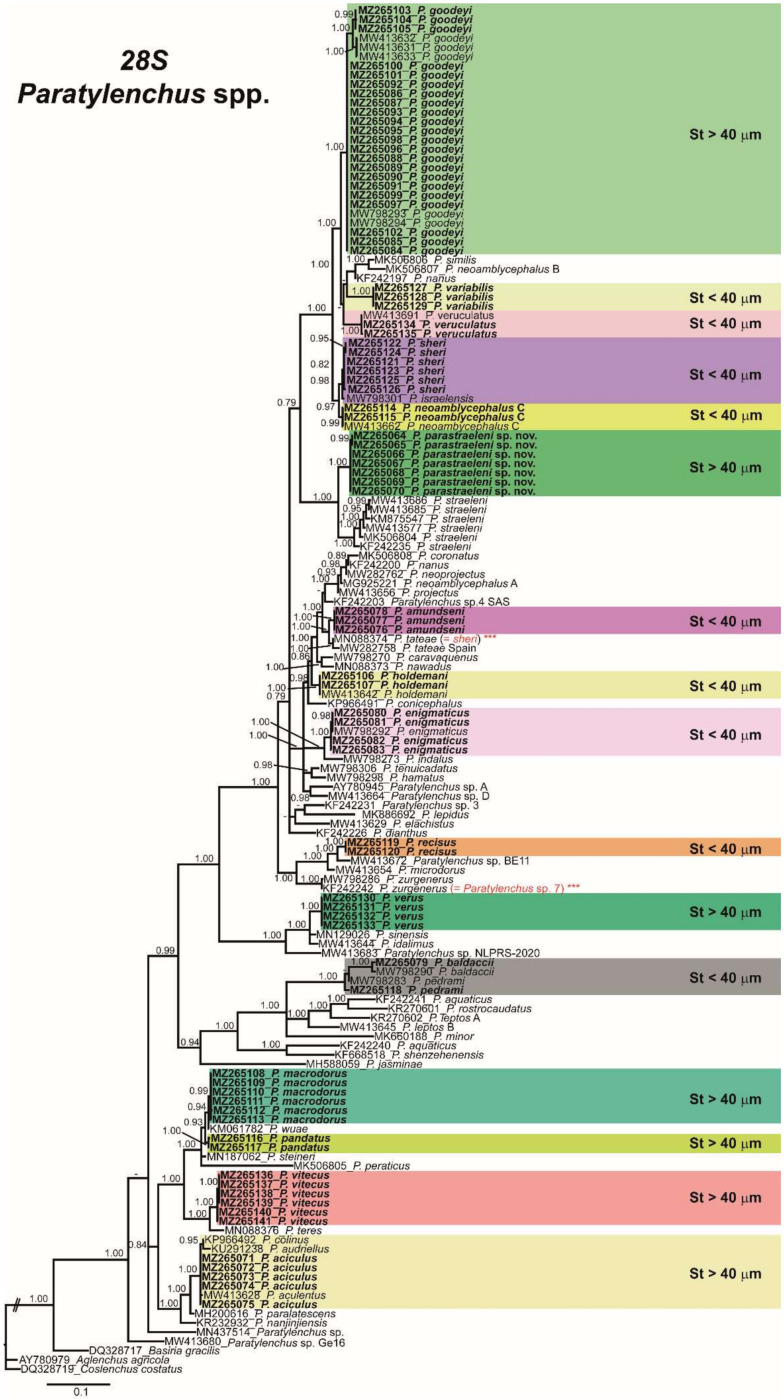
Phylogenetic relationships within the genus *Paratylenchus*. Bayesian 50% majority rule consensus tree as inferred from D2-D3 expansion domains of the 28S rRNA sequence alignment under the general time-reversible model of sequence evolution with correction for invariable sites and a gamma-shaped distribution (GTR + I + G). Posterior probabilities of more than 0.70 are given for appropriate clades. Newly obtained sequences in this study are shown in bold. The scale bar indicates expected changes per site. *** Red font names refer to the previous consideration in NCBI.

The D2-D3 domains of the 28S rRNA gene alignment (702 bp long) included 148 sequences of 64 *Paratylenchus* species and three outgroup species (*Basiria gracillis* (DQ328717), *Aglenchus agricola* (AY780979), and *Coslenchus costatus* (DQ328719)). Seventy-eight new sequences were included in this analysis. The Bayesian 50% majority rule consensus tree inferred from the D2-D3 alignment is given in Figure 16. The tree contained two moderately supported clades (PP = 0.94, PP = 0.84). These clades are mainly coincident with other recent studies on *Paratylenchus* spp. [3,4]. The new species, *P. parastraeleni* sp. nov., clustered with several accessions of *P. straeleni* from Belgium, Iran, South Africa, and Turkey, but clearly separated into two different subclades (PP = 1.00) (Figure 16). Newly sequenced species clustered in separated clusters and subclusters, *viz*. *P. variabilis*, *P. amundseni*, *P. recisus*, *P. verus*, *P. macrodorus*, *P. pandatus*, *P. vitecus* and *P. aciculus*, but with mixed stylet patterns (long and flexible stylet > 40 µm with conus representing about more than 70% of the total stylet and short and rigid stylet < 40 µm with conus about 50% of the total stylet) within the main clusters, except for a basal clade moderately supported (PP = 0.84) comprising 14 species with stylet > 40 µm, including the four species newly sequenced herein (*P. aciculus*, *P. macrodorus*, *P. pandatus*, and *P. vitecus*) (Figure 16).

**Figure 17:**
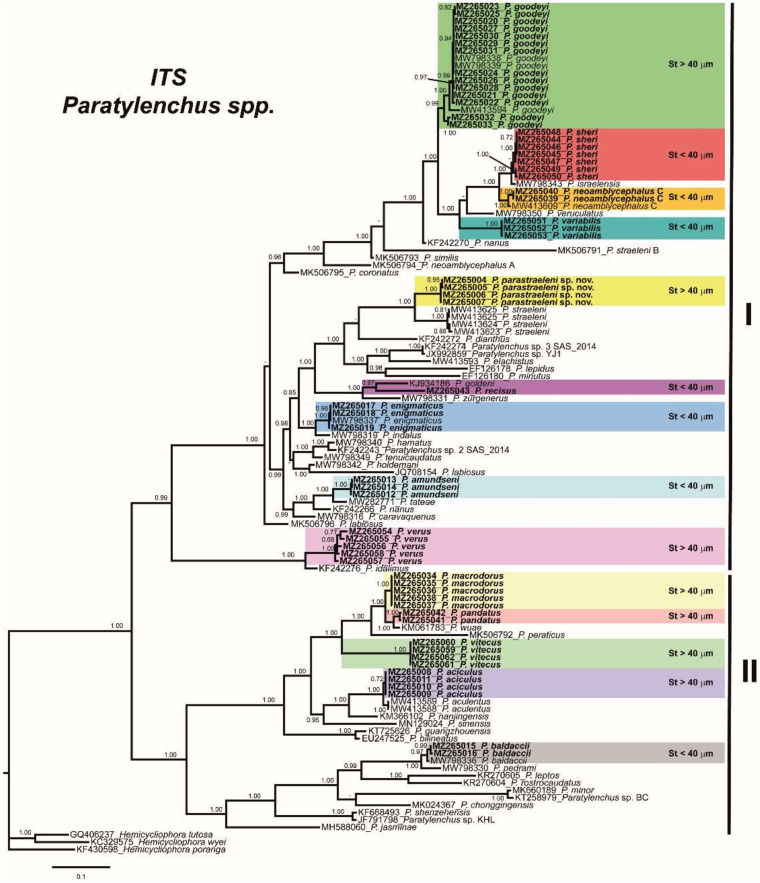
Phylogenetic relationships within the genus *Paratylenchus*. Bayesian 50% majority rule consensus tree as inferred from ITS rRNA sequence alignment under the general time-reversible model of sequence evolution with correction for invariable sites and a gamma-shaped distribution (GTR + I + G). Posterior probabilities of more than 0.70 are given for appropriate clades. Newly obtained sequences in this study are shown in bold. The scale bar indicates expected changes per site.

The ITS rRNA gene alignment (836 bp long) included 117 sequences of 55 *Paratylenchus* species and three outgroup species (*Hemicycliophora lutosa* (GQ406237), *H. wyei* (KC329575) and *H. poranga* (KF430598)). Fifty-nine new sequences were included in this analysis. The Bayesian 50% majority rule consensus tree inferred from the ITS alignment is given in Figure 17. The tree contained two highly supported major clades I and II (PP = 0.99 and PP = 1.00, respectively) and several subclades (Figure 17). Clade I includes mostly species with short stylet (<40 µm), but also species with long stylet (>40 µm), including all isolates of *P. goodeyi*, the new species *P. parastraeleni* sp. nov., *P. straeleni*, *P. verus* and *P. idalimus* (Figure 17). Clade II mostly includes species with long stylet (>40 µm), but also species with short stylet (<40 µm), including *P. baldaccii*, *P. pedrami*, *P. jasminae*, *P. minor*, and *P. rostrocaudatus* (Figure 17). These clades were partially coincident with previous studies with, in some cases, similar or different clade support [3,4].

**Figure 18:**
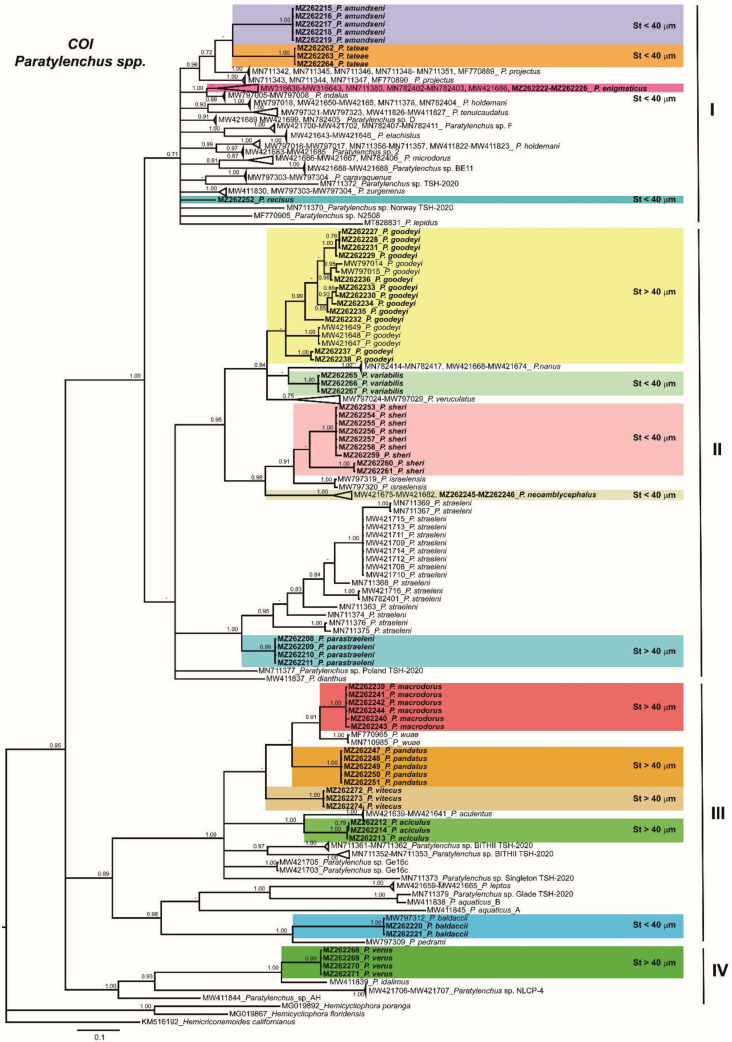
Phylogenetic relationships within the genus *Paratylenchus*. Bayesian 50% majority rule consensus tree as inferred from cytochrome c oxidase subunit 1 (COI) sequence alignment under the general time-reversible model of sequence evolution with a gamma-shaped distribution (GTR + G). Posterior probabilities of more than 0.70 are given for appropriate clades. Newly obtained sequences in this study are shown in bold. The scale bar indicates expected changes per site.

The COI gene alignment (384 bp long) included 245 sequences of 51 *Paratylenchus* species and three outgroup species (Hemicriconemoides californianus (KM516192), *Hemicycliophora floridensis* (MG019867) and *H. poranga* (MG019892)). Sixty-seven new sequences were included in this analysis. The Bayesian 50% majority rule consensus tree inferred from the COI sequence alignment is given in Figure 18. The tree contained four major clades, but only one basal clade (IV) was well supported (PP = 1.00), including two unidentified *Paratylenchus* species and *P. verus* and *P. idalimus*, and all others (clades I, II, and III) low supported (PP < 0.70 to 0.89). The *P. straeleni*-complex clustered in a well-supported subclade (PP = 1.00) within clade II, and the new species, *P. parastraeleni* sp. nov., was clearly separated from all other isolates of *P. straeleni* from Belgium, Canada, Ireland, and USA (Figure 18). Similar as in ribosomal markers, stylet length patterns (> or <40 µm) were mixed in clusters II and III, whereas cluster I comprises species with short stylets and clade IV species with long stylets (Figure 18). These clades were partially coincident with other studies with, in some cases, similar or different clade support [3,4].

## 3. Discussion

This research comprises the second part focused on the integrative taxonomical identification of pin nematodes of the genus *Paratylenchus* in Spain. These results increase the number of species with morphological and molecular data for their unequivocal identification, as well as confirming the huge biodiversity of this group including the description of a new species *viz*. *P. parastraeleni* sp. nov., within the *P. straeleni*-complex.

Eighteen *Paratylenchus* spp. from nine different localities, including almond and natural environment soil samples, were identified. All of them except one, were already known (*P. amundseni*, *P. aciculus*, *P. baldaccii*, *P. enigmaticus*, *P. goodeyi*, *P. holdemani*, *P. macrodorus*, *P. neoamblycephalus*, *P. pandatus*, *P. pedrami*, *P. recisus*, *P. sheri*, *P. tateae*, *P. variabilis*, *P. veruculatus*, *P. verus*, and *P. vitecus*), and eight considered as first reports for Spain in this work (*viz*. *P. amundseni*, *P. aciculus*, *P. neoamblycephalus*, *P. pandatus*, *P. recisus, P. variabilis, P. verus* and *P. vitecus*). Finally, one of the 18 species detected was identified as a new species, *P. parastraeleni* sp. nov., which confirmed the cryptic diversity within the *P. straeleni*-species complex group by applying integrative taxonomical approaches verifying an outstanding example of the cryptic diversity. Overall, the results of this and previous studies reported a total of 39 species of *Paratylenchus* in Spain, widespread in cultivated and natural ecosystems.

In *Paratylenchus* spp. with longer stylet (>40 µm) most juveniles bear elongate flexible stylet (formerly belonging to the genus *Gracilacus*), but some species are found to have what appears to be fourth-stage juveniles with very length reduced and rigid stylets, a characteristic most frequently found in species of *Paratylenchus sensu stricto* with female stylets of 40 µm or less [36]. Since many soil samples from natural environments comprise mixed species (even four different species), it is very difficult to associate specimens of one developmental stage with the appropriate adult state [3,4]. However, applying integrative taxonomical approaches (molecular barcoding of juvenile and adult individuals) we can accurately study juvenile and adult forms in each soil sample. For the first time, morphological and molecular data (D2-D3, ITS and COI for the same individual) of J4 for the majority of the species detected in this study were provided herein, allowing the first report for authenticating a clear example of stylet and lip region metamorphosis between J4 and adult female. Within several isolates of *P. goodeyi* studied here, we verified that short rigid stylet and conoid-truncate lip region with strong labial sclerotization in J4 moved to a long and slender flexible stylet and a conoid-rounded lip region without labial sclerotization in adult females. Apart from the unequivocal identification of juvenile stages of each species, the integrative taxonomical identification of J4 allows to document some important biological aspects for some species, as well as a useful tool for the species identification in periods when the resting-stage accumulates predominantly in soil under adverse environmental conditions (*viz*. drought conditions) [3,4].

Although we are aware that nematological efforts on *Paratylenchus* species in Southern Spain have been higher than that carried out in central and northern parts of the country, the present distribution of the genus in Spain, with about 90% of species (35 out of 39 species, and 24 of them confirmed by integrative taxonomy) only reported in Southern Spain, suggest that this part of the country can be considered as a potential hotspot of biodiversity. Nevertheless, further research is needed to definitely confirm this hypothesis. This study also ratifies the previous proposed hypothesis [4] that we have only deciphered just a small part of the species diversity of pin nematodes reported in Spain, indicating that the biodiversity of this group is far from being adequately explored all over the world [3,4]. The present data also suggest that species richness was higher in natural environments than in cultivated areas, since the number of *Paratylenchus* species detected within the same sample in natural environments included four different species (*viz*. *P. holdemani*, *P. macrodorus*, *P. pedrami*, and *P. veruculatus* in wild olive sample code as AR_102), and more than 60% of soil samples from natural environments exhibited at least two *Paratylenchus* species, whereas in the majority of samples from cultivated areas only one or maximum two mixed species were frequently detected in the same sample [3,4,8]. Nevertheless, this hypothesis needs to be contrasted with further investigations.

*Paratylenchus microdorus* has been extensively reported in Spain in cultivated and natural environments [15,16,17,18]. The present results, comprising integrative studies on some geographical areas with previous records of *P. microdorus*, suggest that probably this species was misidentified in previous records. In this case, *P. recisus*, *P. variabilis*, *P. veruculatus*, and *P. zurgenerus*, are close morphologically to *P. microdorus*, but molecularly well separated. This study suggests that previous records of *P. microdorus* could be misidentified, since only detailed traits can separate these species (short differences in stylet length, shape of tail terminus, vulva anus distance with regard to tail length), and therefore additional studies are needed to clarify the real biodiversity in the *P. microdorus*-species complex in Spain by applying integrative taxonomy. Probably, this potential misidentification can also be referred to the numerous records of *P. microdorus* in other countries such as Bulgaria, Germany, Hungary, Poland, and Romania [2], which need further investigations. Molecularly, *P. microdorus*-species complex was separated in two subgroups, one comprising *P. microdorus*, *P. recisus* and *P. zurgenerus*, and another very separate subclade including *P. variabilis* and *P. veruculatus*, being consistent for ribosomal and mitochondrial genes.

The genus *Paratylenchoides* was proposed by Raski [44] to accommodate two *Paratylenchus* species populations from France and Israel with heavy sclerotization in the lip region and narrow lip region dorso-ventrally. This action was partially followed by Siddiqi [48] proposing a subgeneric rank within the genus *Paratylenchus*. However, Raski and Luc [49] considered that differences between *Paratylenchoides* and *Paratylenchus* were minor and cannot be considered important to separate both taxa, synonymizing *Paratylenchoides* with *Paratylenchus*. The present results confirm that, molecularly, *P. sheri* and *P. israelensis* (formerly *Paratylenchoides* species) clustered together in two separate subclades in D2-D3, ITS and COI trees, but always together with other *Paratylenchus* species with long and short stylets, such as *P. neoamblycephalus*, *P. veruculatus* or *P. parastraeleni* sp. nov. and *P. goodeyi* and cannot be considered a separate genus as it is the case for *Gracilacus* already discussed [3,4,6].

The results obtained in the present study, reinforce the idea that for accurate identification of *Paratylenchus* spp. it is essential to carry out an integrative identification, including morphological, morphometrical and molecular analysis, the latter of which should be based on multilocus approaches (D2-D3 region of 28S rRNA and COI) [3,4,6]. In our case several species demonstrate low differences in ribosomal markers (98–99%) among species, but clear differences on COI and are also clearly different morphologically. This situation has been observed among *P. sheri*–*P. israelensis*–*P. neoamblycephalus, P. macrodorus–P. pandatus–P. wuae* or between *P. aciculus*–*P. aculentus.* This is because mitochondrial DNA display a high mutation rate and maternal inheritance, which also enables better discrimination of closely related species [50,51]. On the other hand, several species showed some molecular intraspecific variability in the three regions studied herein (0.5–4%) but with identical morphology and morphometry, such as *P. goodeyi*, *P. enigmaticus*.

Phylogenetic analyses based on D2-D3, ITS, and COI gene using BI mostly agree with the clustering obtained by other authors [3,4,6]. Ribosomal and mitochondrial phylogenies did not separate the long stylet length (>40 μm) with the short stylet length (>40 μm) supporting the synonymy of *Gracilacus* and suggesting that stylet length in *Paratylenchus* has evolved independently several times during the evolution of this genus [3,4,6].

## 4. Materials and Methods

### 4.1. Nematode Sampling and Morphological Identification

Fifteen soil samples were collected mainly from the rhizosphere of herbaceous and woody plants including 5 samples from almond with different rootstocks (*Prunus* spp.), 2 samples from Portuguese oak (*Quercus faginea* Lam.), 3 samples from Aleppo pine (*Pinus halepensis* Mill.), 3 samples from wild olive (*Olea europaea* sbsp. *silvestris* (Mill.) Lehr), and 2 samples from grasses, in 10 localities in Spain (Table 1). Samples were collected using a shovel and considering the upper 5–40 cm depth of soil. Nematodes were extracted from a 500-cm^3^ subsample of soil by centrifugal flotation [52].

A total of 232 individuals including 160 females, 5 males and 67 juveniles were used for morphological and morphometrical analyses. Specimens for study using light microscopy (LM) and morphometrical studies were killed and fixed in an aqueous solution of 4% formaldehyde + 1% glycerol, dehydrated using alcohol-saturated chamber and processed to pure glycerine using Seinhorst’s method [53] as modified by De Grisse [54]. The developmental stage of the juveniles was determined according to the body length and the degree of development of genital cells [26]. Light micrographs were taken using fresh nematodes and measurements of each nematode population, including important diagnostic characteristics (i.e., de Man indices, body length, stylet length, lip region, tail shape) [55], were performed using a Leica DM6 compound microscope with a Leica DFC7000 T digital camera using fixed and embedded nematodes in glycerin. Nematodes were identified at the species level using an integrative approach combining molecular and morphological techniques to achieve efficient and accurate identification [3,4,8]. For each nematode population, key diagnostic characters were determined, including body length, stylet length, a ratio (body length/maximum body width), b ratio (body length/total pharynx length), c ratio (body length/tail length), c’ ratio (tail length/body width at anus), V ratio ((distance from anterior end to vulva/body length) × 100), and o ratio ((distance from stylet base to dorsal pharyngeal opening/stylet length) × 100) [3,4,8], and the sequencing of specific DNA fragments (described below) confirmed the identity of the nematode species for each population. Specimens for SEM observations were processed using Wergin’s method [56], coated with gold and observed with a JEOL 50A scanning electron microscope at 10 kV of accelerating voltage.

Nematode populations of *Paratylenchus* species already described were analyzed morphologically and molecularly in this study and proposed as standard and reference populations for each species given until topotype material becomes available and molecularly characterized. Voucher specimens of these described species have been deposited in the nematode collection of Institute for Sustainable Agriculture, IAS-CSIC, Córdoba, Spain.

### 4.2. Nematode Molecular Characterization

For molecular analyses, and in order to avoid mistakes in case of mixed populations in the same sample (being common in several soil samples), single specimens from the sample were temporarily mounted in a drop of 1 M NaCl containing glass beads (to avoid nematode crushing/damaging specimens) to ensure specimens conformed with the unidentified population. All necessary morphological and morphometrical data by taking pictures and measurements using the above camera-equipped microscope were recorded. Then DNA extraction from single individuals was performed as described by Palomares-Rius et al. [57], and more importantly, for all the 24 studied isolates, all the three molecular markers of each *Paratylenchus* isolate belong to the same single extracted individual in each PCR tube without any exception. In addition, male and juveniles conspecificity was proven by single DNA extraction of male or juveniles for each species.

The D2 and D3 expansion domains of the 28S rRNA were amplified using the D2A (5′-ACAAGTACCGTGAGGGAAAGTTG-3′) and D3B (5′-TCGGAAGGAACCAGCTACTA-3′) primers [58]. The Internal Transcribed Spacer region (ITS) was amplified by using forward primer TW81 (5′- GTTTCCGTAGGTGAACCTGC -3′) and reverse primer AB28 (5′-ATATGCTTAAGTTCAGCGGGT -3′) [59]. The COI gene was amplified using the primers JB3 (5′-TTTTTTGGGCATCCTGAGGTTTAT-3′) and JB5 (5′-AGCACCTAAACTTAAAACATAATGAAAATG-3′) [60]. The PCR cycling conditions for the 28S rRNA and ITS regions were as follows: 95 °C for 15 min, followed by 35 cycles of 94 °C for 30 s, an annealing temperature of 55 °C for 45 s, and 72 °C for 1 min, and one final cycle of 72 °C for 10 min. The PCR cycling for COI primers was as follows: 95 °C for 15 min, 39 cycles at 94 °C for 30 s, 53 °C for 30 s, and 68 °C for 1 min, followed by a final extension at 72 °C for 7 min. PCR volumes were adapted to 25 μL for each reaction, and primer concentrations were as described in De Ley et al. [58], Subbotin et al., [59] and Bowles et al. [60]. We used 5× HOT FIREpol Blend Master Mix (Solis Biodyne, Tartu, Estonia) in all PCR reactions. The PCR products were purified using ExoSAP-IT (Affimetrix, USB products, Kandel, Germany) and used for direct sequencing in both directions with the corresponding primers. The resulting products were run in a DNA multicapillary sequencer (Model 3130XL Genetic Analyzer; Applied Biosystems, Foster City, CA, USA), using the BigDye Terminator Sequencing Kit v.3.1 (Applied Bio-systems) at the Stab Vida sequencing facility (Caparica, Portugal). The sequence chromatograms of the 3 markers (ITS, COI and D2-D3 expansion segments of 28S rRNA) were analyzed using DNASTAR LASERGENE SeqMan v. 7.1.0. Basic local alignment search tool (BLAST) at the National Center for Biotechnology Information (NCBI) was used to confirm the species identity of the DNA sequences obtained in this study [61]. The newly obtained sequences were deposited in the GenBank database under accession numbers indicated on the phylogenetic trees and in Table 1.

### 4.3. Phylogenetic Analyses

D2-D3 expansion segments of 28S rRNA, ITS rRNA, and COI mtDNA sequences of the 24 *Paratylenchus* isolates were obtained in this study. These sequences and other sequences from species of *Paratylenchus* from GenBank were used for phylogenetic analyses. Selection of outgroup taxa for each dataset were based on previously published studies [3,4,7,62]. Multiple sequence alignments of the different genes were completed using the FFT-NS-2 algorithm of MAFFT V.7.450 [63]. BioEdit program V. 7.2.5 [64] was used for sequence alignments visualization and edited by Gblocks ver. 0.91b [65] in Castresana Laboratory server (http://molevol.cmima.csic.es/castresana/Gblocks_server.html accessed on 13 May 2021) using options for a less stringent selection (minimum number of sequences for a conserved or a flanking position: 50% of the number of sequences +1; maximum number of contiguous non-conserved positions: 8; minimum length of a block: 5; allowed gap positions: with half). Phylogenetic analyses of the sequence datasets were based on Bayesian inference (BI) using MrBayes 3.1.2 [66]. The best-fit model of DNA evolution was achieved using JModelTest V.2.1.7 [67] with the Akaike information criterion (AIC). The best-fit model, the base frequency, the proportion of invariable sites, and the gamma distribution shape parameters and substitution rates in the AIC were then used in MrBayes for the phylogenetic analyses. The general time-reversible model with invariable sites and a gamma-shaped distribution (GTR + I + G) for the D2-D3 segments of 28S rRNA and the partial ITS rRNA and the general time-reversible model with a gamma-shaped distribution (GTR + G) for COI gene, were run with four chains for 4, 4, and 10 × 10^6^ generations, respectively. A combined analysis of the three ribosomal genes was not undertaken due to some sequences not being available for all species. The sampling for Markov chains was carried out at intervals of 100 generations. For each analysis, two runs were conducted. After discarding burn-in samples of 30% and evaluating convergence, the remaining samples were retained for more in-depth analyses. The topologies were used to generate a 50% majority-rule consensus tree. On each appropriate clade, posterior probabilities (PP) were given. FigTree software version v.1.42 [68] was used for visualizing trees from all analyses.

## 5. Conclusions

This study reveals the existence of a huge cryptic biodiversity within the genus *Paratylenchus*, increasing and expanding the diversity of this group in Spain. For the first time, morphological and molecular data (D2-D3, ITS and COI for the same individual) of J4 allowed to authenticate an example of stylet and lip region metamorphosis between J4 and adult females in *P. goodeyi* (from short rigid stylet and conoid-truncate lip region with strong labial sclerotization in J4 to a long and slender flexible stylet and a conoid-rounded lip region without labial sclerotization in adult females). This study also ratifies the previous proposed hypothesis that we have only deciphered just a small part of the species diversity within pin nematodes reported in Spain and most probably all over the world. Our data also suggest that *P. microdorus* comprise a complex of species morphologically very close, but molecularly well separated, and therefore additional studies are needed to clarify the real biodiversity within the *P. microdorus*-species complex in Spain and all over the world by applying integrative taxonomy.

## Figures and Tables

**Table 1:** Isolates sampled and sequenced for *Paratylenchus* spp. from several localities in Spain used in this study.

Species	Sample Code	Locality, Province, Host	D2-D3	ITS	*COI*
1. *P. parastraeleni* sp. nov.	CAZ_05	Arroyo Frío, Jaén, *Quercus faginea* Lam.	MZ265064-MZ265070	MZ265004-MZ265007	MZ262208-MZ262211
2. *P. aciculus* Brown, 1959	CAZ_07	Coto Ríos, Jaén, *Pinus halepensis* Mill.	MZ265071-MZ265075	MZ265008-MZ265011	MZ262212-MZ262214
3. *P. amundseni* Bernard, 1982	CAZ_02	La Iruela, Jaén, *Pinus halepensis* Mill.	MZ265076-MZ265078	MZ265012-MZ265014	MZ262215-MZ262219
4. *P. baldaccii* (Oostenbrink, 1953) Raski, 1962	CAZ_04	Arroyo Frío, Jaén, grasses	MZ265079	MZ265015-MZ265016	MZ262220-MZ262221
5. *P. enigmaticus* Munawar et al., 2021	IAS_21	Córdoba, Córdoba, grasses	MZ265080-MZ265083	MZ265017-MZ265019	MZ262222-MZ262226
6. *P. goodeyi* Oostenbrink, 1953	EP_ACA	Córdoba, Córdoba, wild olive	MZ265084-MZ265091	MZ265020-MZ265024	MZ262227-MZ262230
	AR_097	Santa Mª de Trasierra, Córdoba, wild olive	MZ265092-MZ265097	MZ265025-MZ265026	MZ262231-MZ262233
	PR_050	Montalbán, Córdoba, almond	MZ265098-MZ265099	MZ265027	MZ262234-MZ262235
	PR_017	Córdoba, Córdoba, almond	MZ265100	MZ265028	MZ262236
	PR_076	Marmolejo, Jaén, almond	MZ265101-MZ265102	MZ265029-MZ265031	- *
	PR_019	Córdoba, Córdoba, almond	MZ265103-MZ265105	MZ265032-MZ265033	MZ262237-MZ262238
7. *P. holdemani* Raski, 1975	AR_102	Santa Mª de Trasierra, Córdoba, wild olive	MZ265106-MZ265107	-	-
8. *P. macrodorus* Brzeski, 1963	AR_102	Santa Mª de Trasierra, Córdoba, wild olive	MZ265108-MZ265113	MZ265034-MZ265038	MZ262239-MZ262244
9. *P. neoamblycephalus* Geraert, 1965	CAZ_05	Arroyo Frío, Jaén, *Quercus faginea* Lam.	MZ265114-MZ265115	MZ265039-MZ265040	MZ262245-MZ262246
10. *P. pandatus* (Raski, 1976) Siddiqi, 1986	PIN_AR	Caravaca, Murcia, *Pinus halepensis* Mill.	MZ265116-MZ265117	MZ265041-MZ265042	MZ262247-MZ262251
11. *P. pedrami* Clavero-Camacho et al., 2021	AR_102	Santa Mª de Trasierra Córdoba, wild olive	MZ265118	-	-
12. *P. recisus* Siddiqi, 1996	CAZ_06	Arroyo Frío, Jaén, *Quercus faginea* Lam.	MZ265119-MZ265120	MZ265043	MZ262252
13. *P. sheri* (Raski, 1973) Siddiqi, 1986	CAZ_04	Arroyo Frío, Jaén, grasses	MZ265121-MZ265124	MZ265044-MZ265048	MZ262253-MZ262259
	CAZ_07	Coto Ríos, Jaén, *Pinus halepensis* Mill.	MZ265125-MZ265126	MZ265049-MZ265050	MZ262260-MZ262261
14. *P. tateae* Wu and Townshend (1973)	PR_187	Ariza, Zaragoza, almond	MW282754-MW282759	MW282766-MW282771	MZ262262-MZ262264
15. *P. variabilis* Raski, 1975	EP_ACA	Córdoba, Córdoba, wild olive	MZ265127-MZ265129	MZ265051-MZ265053	MZ262265-MZ262267
16. *P. veruculatus* Wu, 1962	AR_102	Santa Mª de Trasierra Córdoba, wild olive	MZ265134-MZ265135	-	-
17. *P. verus* (Brzeski, 1995) Brzeski, 1998	AR_097	Santa Mª de Trasierra, Córdoba, wild olive	MZ265130-MZ265133	MZ265054-MZ265058	MZ262268-MZ262271
18. *P. vitecus* (Pramodini et al., 2006) Ghaderi et al., 2014	EP_ACA	Córdoba, Córdoba, wild olive	MZ265136-MZ265141	MZ265059-MZ265062	MZ262272-MZ262274

^*^ = not sequenced.

**Table 2:** Morphometrics of *Paratylenchus parastraeleni* sp. nov. paratype females, males and fourth-stage juveniles. All measurements are in µm and in the form: mean ± s.d. (range).

	Holotype	Paratypes		
	Female	Females	Males	Juveniles (J4)
Sample Code	CAZ_05	CAZ_05	CAZ_05	CAZ_05
Locality	Arroyo Frío, Jaén			
n	1	19	4	5
L	425	417.7 ± 35.2 (363–467)	389.5 ± 19.3 (369–414)	337.6 ± 37.5 (302–382)
a *	23.6	20.6 ± 3.4 (16.3–26.4)	28.9 ± 1.3 (27.3–30.3)	22.2 ± 1.2 (20.8–23.9)
b	3.5	3.6 ± 0.3 (3.1–4.2)	4.2 ± 0.7 (3.6–5.2)	3.3 ± 0.4 (2.9–3.8)
c	14.7	13.4 ± 1.5 (11.4–16.5)	12.7 ± 1.2 (11.9–14.5)	12.9 ± 1.9 (10.7–15.9)
c’	2.6	2.9 ± 0.2 (2.5–3.4)	2.9 ± 0.1 (2.7–3.0)	2.8 ± 0.3 (2.4–3.2)
V or T	80.9	82.1 ± 0.9 (80.2–83.5)	-	-
G1	35.5	44.4 ± 4.4 (35.5–50.3)	-	-
Stylet length	54.0	53.5 ± 1.5 (52.0–56.0)	-	45.8 ± 2.2 (43.0–48.0)
(Stylet length/body length) × 100	12.7	12.9 ± 0.9 (11.3–14.6)	-	13.7 ± 1.0 (12.6–14.7)
Conus length	43.0	41.2 ± 1.4 (38.0–43.0)	-	36.0 ± 3.2 (31.0–39.0)
m	79.6	77.1 ± 1.9 (73.1–79.6)	-	78.6 ± 6.3 (72.1–88.6)
DGO	5.5	5.4 ± 0.5 (4.5–6.0)	-	4.0 ± 0.7 (3.0–5.0)
O	10.2	10.2 ± 1.0 (8.0–11.5)	-	8.7 ± 1.4 (6.8–10.6)
Lip width	5.5	4.9 ± 0.4 (4.0–5.5)	4.1 ± 0.3 (4.0–4.5)	4.8 ± 0.4 (4.5–5.0)
Median bulb length	24.0	24.0 ± 2.7 (19.0–29.0)	-	23.8 ± 2.8 (19.0–26.0)
Median bulb width	11.0	11.1 ± 0.9 (9.0–13.0)	-	9.4 ± 1.0 (8.5–11.0)
Anterior end to center median bulb	78	74.0 ± 3.8 (67.0–81.0)	-	62.4 ± 4.0 (58.0–68.0)
MB	65.0	64.2 ± 2.5 (58.5–70.0)	-	61.3 ± 4.0 (56.9–67.3)
Nerve ring to anterior end	88.0	89.4 ± 5.1 (77.0–98.0)	-	78.8 ± 4.2 (74.0–84.0)
Excretory pore to anterior end	94.0	98.2 ± 6.6 (87.0–114.0)	81.5 ± 5.7 (74.0–88.0)	86.4 ± 6.1 (77.0–94.0)
Pharynx length	120.0	115.2 ± 4.9 (107.0–123.0)	95.0 ± 11.1 (80.0–106.0)	101.8 ± 0.8 (101.0–103.0)
Maximum body diam.	18.0	20.9 ± 4.3 (14.0–28.0)	13.2 ± 0.3 (13.0–13.5)	15.2 ± 1.8 (14.0–18.0)
Tail length	29.0	31.4 ± 4.1 (25.5–39.0)	30.9 ± 3.7 (25.5–34.0)	26.8 ± 5.9 (19.5–35.0)
Anal body diam.	11.0	11.0 ± 1.4 (8.5–14.0)	10.6 ± 0.9 (9.5–11.5)	9.7 ± 1.7 (8.0–12.0)
Spicules	-	-	22.0 ± 1.1 (21.0–23.5)	-
Gubernaculum	-	-	4.0 ± 0.4 (3.5–4.5)	-

* Abbreviations: a = body length/greatest body diameter; b = body length/distance from anterior end to pharyngo-intestinal junction; DGO = distance between stylet base and orifice of dorsal pharyngeal gland; c = body length/tail length; c’ = tail length/tail diameter at anus or cloaca; G1 = anterior genital branch length expressed as percentage (%) of the body length; L = overall body length; m = length of conus as percentage of total stylet length; MB = distance between anterior end of body and center of median pharyngeal bulb expressed as percentage (%) of the pharynx length; n = number of specimens on which measurements are based; O = DGO as percentage of stylet length; T = distance from cloacal aperture to anterior end of testis expressed as percentage (%) of the body length; V = distance from body anterior end to vulva expressed as percentage (%) of the body length.

**Table 3:** Morphometrics of *Paratylenchus aciculus* Brown, 1959 from Coto Ríos, Jaén, Spain, type population, and *P. aculentus* from Belgium. All measurements are in µm and in the form: mean ± s.d. (range).

Species	*P. aciculus*	*P. aciculus*	*P. aculentus*
Life Stage	Females	Females	Females
Sample Code	CAZ_07	Type	Belgium
Locality, Province	Coto Ríos, Jaén	Population [31]	Singh et al. [3]
n	12	25	12
L	309.3 ± 16.2 (285–339)	240–310	266 ± 20.1 (233–03)
a *	19.1 ± 1.7 (17.0–21.5)	18–24	19.6 ± 2.0 (16.3–23.2)
b	2.5 ± 0.2 (2.2–2.8)	2.4–2.7	2.6 ± 0.1 (2.4–2.8)
c	12.4 ± 1.4 (10.3–14.8)	10–16	12.4 ± 1.5 (10.8–15.2)
c’	2.9 ± 0.2 (2.6–3.5)	3.0	2.8 ± 0.3 (2.4–3.1)
V	73.4 ± 0.8 (72.3–74.7)	68–74	72.5 ± 1.5 (70.8–75.7)
G1	34.8 ± 7.6 (23.5–42.5)	-	-
Stylet length	71.4 ± 2.8 (67.5–75.0)	61–69	56.0 ± 3.3 (52.4–61.2)
(Stylet length/body length) × 100	23.1 ± 1.0 (21.8–24.6)	-	-
Conus length	64.7 ± 3.5 (58.0–69.0)	-	49.1 ± 3.6 (43.0–54.9)
m	90.6 ± 2.7 (84.1–93.2)	-	-
DGO	5.3 ± 0.6 (4.5–6.5)	-	-
O	7.4 ± 0.7 (6.3–8.7)	-	-
Lip width	5.4 ± 0.5 (5.5–6.5)	-	-
Median bulb length	24.3 ± 1.9 (22.0–27.0)	-	-
Median bulb width	11.5 ± 0.5 (11.0–12.0)	-	-
Anterior end to center median bulb	85.0 ± 3.3 (79.0–89.0)	-	-
MB	66.2 ± 2.5 (62.3–69.0)	-	-
Nerve ring to anterior end	103.2 ± 4.7 (94.0–100.0)	-	-
Excretory pore to anterior end	83.8 ± 5.9 (72.5–91.0)	70	66.7 ± 5.2 (54.3–74.4)
Pharynx length	125.5 ± 8.2 (109.0–138.0)		101 ± 8.3 (87.0–113)
Maximum body diam.	16.4 ± 2.1 (14.0–20.0)	15	13.6 ± 1.3 (11.6–15.5)
Tail length	25.3 ± 3.4 (20.5–33.0)	50	20.9 ± 2.3 (18.1–25.1)
Anal body diam.	8.6 ± 0.7 (7.5–10.0)	-	7.6 ± 0.5 (7.0–8.3)

* Abbreviations: a = body length/greatest body diameter; b = body length/distance from anterior end to pharyngo-intestinal junction; DGO = distance between stylet base and orifice of dorsal pharyngeal gland; c = body length/tail length; c’ = tail length/tail diameter at anus or cloaca; G1 = anterior genital branch length expressed as percentage (%) of the body length; L = overall body length; m = length of conus as percentage of total stylet length; MB = distance between anterior end of body and center of median pharyngeal bulb expressed as percentage (%) of the pharynx length; n = number of specimens on which measurements are based; O = DGO as percentage of stylet length; V = distance from body anterior end to vulva expressed as percentage (%) of the body length.

**Table 4:** Morphometrics of *Paratylenchus* *amundseni* Bernard, 1982 from La Iruela, Jaén, Spain. All measurements are in µm and in the form: mean ± s.d. (range).

Life Stage	Females	Fourth-Stage Juveniles	Females
Sample Code	CAZ_02	CAZ_02	Type
Locality, Province	La Iruela, Jaén	La Iruela, Jaén	Population [32]
n	17	6	16
L	397.1 ± 35.0 (335–450)	358.7 ± 10.8 (340–369)	320–370
a *	18.7 ± 1.7 (16.0–22.5)	19.0 ± 2.7 (15.6–21.7)	19–25
b	4.2 ± 0.4 (3.6–5.2)	3.8 ± 0.2 (3.6–4.1)	3.6–4.6
c	11.5 ± 2.0 (7.9–16.0)	13.5 ± 2.0 (11.7–17.3)	9–14
c’	3.5 ± 0.4 (2.8–4.4)	2.7 ± 0.4 (2.3–3.2)	4.5
V	80.5 ± 1.4 (78.6–82.8)	-	76–80
G1	45.1 ± 5.6 (35.2–56.7)	-	-
Stylet length	17.0 ± 0.5 (16.0–18.0)	15.7 ± 0.8 (14.0–16.0)	17–19
(Stylet length/body length) × 100	4.3 ± 0.4 (3.8–5.1)	4.4 ± 0.1 (4.1–4.5)	-
Conus length	10.8 ± 0.7 (10.0–12.0)	10.5 ± 0.6 (9.5–11.0)	-
m	63.7 ± 3.4 (58.8–70.6)	67.0 ± 2.5 (62.5–68.8)	-
DGO	4.9 ± 0.6 (4.0–6.0)	4.3 ± 0.5 (3.5–5.0)	-
O	28.5 ± 3.2 (23.5–36.4)	27.6 ± 2.4 (25.0–31.3)	-
Lip width	4.4 ± 0.5 (4.0–5.5)	5.0 ± 0.6 (4.0–5.5)	-
Median bulb length	22.8 ± 2.0 (19.0–25.0)	20.4 ± 0.5 (20.0–21.0)	-
Median bulb width	11.1 ± 1.6 (9.5–14.0)	8.1 ± 0.2 (8.0–8.5)	-
Anterior end to center median bulb	51.1 ± 4.2 (43.0–59.0)	46.8 ± 1.4 (44.0–48.0)	-
MB	53.8 ± 3.1 (46.2–59.8)	50.1 ± 3.5 (44.9–53.9)	-
Nerve ring to anterior end	69.8 ± 5.4 (57.0–81.0)	67.5 ± 4.7 (62.0–74.0)	-
Excretory pore to anterior end	82.8 ± 5.2 (72.0–94.0)	85.0 ± 4.7 (79.0–91.0)	75
Pharynx length	93.8 ± 6.6 (76.0–104.0)	93.5 ± 5.0 (89.0–101.0)	-
Maximum body diam.	21.5 ± 2.9 (16.5–26.0)	19.2 ± 2.9 (16.0–23.0)	18
Tail length	35.5 ± 6.0 (22.0–43.0)	26.9 ± 3.1 (21.0–29.0)	34
Anal body diam.	10.3 ± 1.5 (8.0–13.5)	10.0 ± 1.5 (9.0–12.0)	-

* Abbreviations: a = body length/greatest body diameter; b = body length/distance from anterior end to pharyngo-intestinal junction; DGO = distance between stylet base and orifice of dorsal pharyngeal gland; c = body length/tail length; c’ = tail length/tail diameter at anus or cloaca; G1 = anterior genital branch length expressed as percentage (%) of the body length; L = overall body length; m = length of conus as percentage of total stylet length; MB = distance between anterior end of body and center of median pharyngeal bulb expressed as percentage (%) of the pharynx length; n = number of specimens on which measurements are based; O = DGO as percentage of stylet length; V = distance from body anterior end to vulva expressed as percentage (%) of the body length.

**Table 5:** Morphometrics of *Paratylenchus baldaccii* (Oostenbrink, 1953) Raski, 1962 and *Paratylenchus enigmaticus* Munawar, Yevtushenko, Palomares-Rius and Castillo, 2021, *Paratylenchus holdemani* Raski, 1975, and *Paratylenchus neoamblycephalus* Geraert, 1965 from several localities in Spain. All measurements are in µm and in the form: mean ± s.d. (range).

	*P. baldaccii*	*P. enigmaticus*	*P. holdemani*	*P. neoamblycephalus*
Life Stage	Females	Females	Females	Female
Sample Code	CAZ_04	IAS_21	AR_102	CAZ_05
Locality, Province	Arroyo Frío, Jaén	Córdoba, Córdoba	St. Mª Trasierra, Córdoba	Arroyo Frío, Jaén
n	4	4	4	1
L	270.5 ± 5.2 (267–278)	367.0 ± 11.3 (358–383)	392.3 ± 30.2 (364–435)	363
a *	17.8 ± 2.1 (15.4–20.5)	21.5 ± 1.5 (20.2–23.2)	24.9 ± 1.6 (23.5–27.2)	18.2
b	3.4 ± 0.1 (3.3–3.5)	3.8 ± 0.2 (3.7–4.0)	3.9 ± 0.3 (3.6–4.1)	4.4
c	10.5 ± 1.3 (8.7–11.7)	15.0 ± 1.2 (13.2–16.0)	13.3 ± 1.0 (12.2–14.6)	14.0
c’	2.8 ± 0.1 (2.7–2.9)	2.5 ± 0.2 (2.3–2.8)	3.0 ± 0.4 (2.7–3.5)	2.2
V	81.2 ± 1.5 (79.5–83.0)	83.2 ± 1.2 (81.8–84.5)	81.0 ± 0.8 (79.9–81.9)	81.3
G1	40.1 ± 5.4 (34.5–46.4)	41.8 ± 9.3 (31.8–51.2)	28.4 ± 0.5 (28.0–28.7)	36.9
Stylet length	29.5 ± 1.2 (28.0–31.0)	26.8 ± 1.0 (26.0–28.0)	26.8 ± 0.6 (26.0–27.5)	33.0
(Stylet length/body length) × 100	10.9 ± 0.5 (10.5–11.6)	7.3 ± 0.1 (7.2–7.4)	6.8 ± 0.5 (6.3–7.4)	9.1
Conus length	21.4 ± 1.1 (20.5–23.0)	18.3 ± 0.5 (18.0–19.0)	16.0 ± 0.7 (15.0–16.5)	23
m	72.5 ± 4.8 (67.4–78.0)	68.3 ± 3.8 (64.3–73.1)	59.8 ± 1.5 (57.7–61.1)	69.7
DGO	5.0 ± 0.7 (4.0–5.5)	5.8 ± 0.9 (4.5–6.5)	6.3 ± 0.6 (5.5–7.0)	5.5
O	17.0 ± 2.5 (13.6–19.6)	21.5 ± 3.6 (16.7–25.0)	23.3 ± 2.0 (21.2–25.5)	16.7
Lip width	3.9 ± 0.3 (3.5–4.0)	6.3 ± 0.3 (6.0–6.5)	6.6 ± 0.5 (6.0–7.0)	6
Median bulb length	17.2 ± 0.8 (16.5–18.0)	23.3 ± 3.8 (20.0–27.0)	19.4 ± 3.1 (16.5–23.5)	26
Median bulb width	8.5 ± 0.5 (8.0–9.0)	10.0 ± 0.7 (9.5–11.0)	10.0 ± 0.4 (9.5–10.5)	11
Anterior end to center median bulb	44.8 ± 1.9 (42.0–46.0)	53.5 ± 1.3 (52.0–55.0)	56.5 ± 3.3 (52.5–60.5)	57
MB	56.4 ± 3.7 (52.1–59.7)	55.5 ± 1.1 (54.6–57.1)	56.4 ± 1.0 (55.2–57.6)	69.5
Nerve ring to anterior end	58.3 ± 1.5 (57.0–60.0)	73.3 ± 3.4 (70.0–78.0)	72.8 ± 3.6 (68.0–75.5)	66.0
Excretory pore to anterior end	68.3 ± 3.6 (63.0–71.0)	82.3 ± 4.3 (76.0–83.0)	85.5 ± 5.2 (79.5–82.0)	74.0
Pharynx length	79.5 ± 2.1 (77.0–82.0)	96.5 ± 3.9 (91.0–100.0)	100.3 ± 5.1 (93.0–105.0)	82.0
Maximum body diam.	15.4 ± 2.1 (13.0–18.0)	17.1 ± 1.7 (15.5–19.0)	15.8 ± 0.6 (15.0–16.5)	20.0
Tail length	26.3 ± 4.0 (23.0–32.0)	24.6 ± 2.9 (23.0–29.0)	29.6 ± 3.4 (26.5–33.5)	26.0
Anal body diam.	9.4 ± 1.1 (8.5–11.0)	9.8 ± 0.6 (9.0–10.5)	9.8 ± 0.3 (9.5–10.0)	12.0

* Abbreviations: a = body length/greatest body diameter; b = body length/distance from anterior end to pharyngo-intestinal junction; DGO = distance between stylet base and orifice of dorsal pharyngeal gland; c = body length/tail length; c’ = tail length/tail diameter at anus or cloaca; G1 = anterior genital branch length expressed as percentage (%) of the body length; L = overall body length; m = length of conus as percentage of total stylet length; MB = distance between anterior end of body and center of median pharyngeal bulb expressed as percentage (%) of the pharynx length; n = number of specimens on which measurements are based; O = DGO as percentage of stylet length; V = distance from body anterior end to vulva expressed as percentage (%) of the body length.

**Table 6:** Morphometrics of *Paratylenchus* *goodeyi* Oostenbrink, 1953 from several localities in Spain. All measurements are in µm and in the form: mean ± s.d. (range).

Locality, Province	Córdoba, Córdoba		Sta. Mª Trasierra, Córdoba		Montalbán, Córdoba		Córdoba, Córdoba	
Life Stage	Females	Fourth-Stage Juveniles	Females	Fourth-Stage Juveniles	Females	Fourth-Stage Juveniles	Females	Fourth-Stage Juveniles
Sample Code	EP_ACA	EP_ACA	AR_097	AR_097	PR_050	PR_050	PR_017	PR_017
n	14	10	5	5	4	4	5	5
L	433.8 ± 38.3 (396–513)	440.8 ± 25.3 (409–486)	466.4 ± 35.1 (408–495)	432.8 ± 19.0 (412–461)	460.0 ± 34.6 (411–490)	395.8 ± 15.0 (375–409)	413.3 ± 8.2 (403–422)	395.8 ± 15.0 (375–409)
a *	22.6 ± 2.5 (18.8–26.8)	23.9 ± 1.5 (21.6–25.8)	22.3 ± 2.2 (19.6–24.3)	24.1 ± 1.6 (21.9–25.8)	22.5 ± 1.9 (20.6–24.3)	22.4 ± 1.9 (20.2–24.7)	20.3 ± 2.0 (17.5–22.2)	22.4 ± 1.8 (20.2–24.7)
b	3.9 ± 0.2 (3.5–4.3)	4.9 ± 0.3 (4.4–5.4)	3.9 ± 0.2 (3.6–4.3)	4.9 ± 0.3 (4.5–5.2)	4.0 ± 0.3 (3.6–4.3)	4.5 ± 0.4 (4.0–4.8)	3.6 ± 0.4 (3.3–4.2)	4.5 ± 0.4 (4.0–4.8)
c	12.6 ± 2.6 (10.7–20.9)	11.2 ± 1.0 (10.0–13.5)	13.7 ± 3.4 (11.3–19.3)	11.4 ± 1.1 (10.1–13.1)	13.4 ± 3.2 (11.6–18.1)	10.8 ± 1.3 (9.4–12.4)	12.0 ± 2.0 (10.6–14.9)	11.1 ± 1.5 (9.9–13.2)
c’	3.5 ± 0.5 (2.1–4.4)	3.2 ± 0.3 (2.6–3.5)	3.3 ± 0.7 (2.3–4.3)	3.0 ± 0.2 (2.7–3.3)	3.3 ± 0.4 (2.7–3.6)	3.1 ± 0.2 (2.8–3.3)	3.3 ± 0.4 (2.7–3.6)	3.0 ± 0.3 (2.6–3.3)
V	80.8 ± 1.3 (78.2–82.4)	-	79.7 ± 1.5 (77.8–81.2)	-	79.2 ± 1.4 (77.8–81.1)	-	80.9 ± 0.9 (79.7–81.8)	-
G1	30.6 ± 2.5 (25.7–33.9)	-	30.8 ± 3.4 (26.1–34.7)	-	29.8 ± 3.0 (26.1–33.1)	-	33.0 ± 2.3 (31.1–36.3)	-
Stylet length	50.9 ± 2.9 (46.0–56.0)	17.4 ± 1.2 (15.0–18.5)	52.8 ± 2.5 (51.0–56.0)	16.9 ± 0.9 (16.0–18.0)	51.8 ± 2.2 (50.0–55.0)	17.1 ± 0.9 (16.0–18.0)	51.8 ± 2.2 (50.0–55.0)	16.9 ± 0.6 (16.0–17.5)
(Stylet length/body length) × 100	11.8 ± 0.7 (10.4–12.7)	4.0 ± 0.3 (3.5–4.4)	11.4 ± 0.8 (10.4–12.5)	3.9 ± 0.3 (3.5–4.4)	11.3 ± 0.9 (10.2–12.4)	4.3 ± 0.3 (3.9–4.7)	12.5 ± 0.5 (12.1–13.2)	4.3 ± 0.3 (3.9–4.7)
Conus length	40.8 ± 2.4 (37.0–45.0)	12.4 ± 1.1 (10.0–14.0)	41.8 ± 1.9 (40.0–45.0)	12.2 ± 1.3 (10.0–13.0)	41.5 ± 1.7 (40.0–44.0)	12.8 ± 0.5 (12.0–13.0)	41.8 ± 2.2 (40.0–45.0)	12.8 ± 0.5 (12.0–13.0)
m	80.2 ± 2.1 (75.0–83.0)	71.3 ± 4.6 (62.5–80.0)	79.2 ± 2.6 (75.0–81.8)	72.1 ± 5.6 (62.5–76.5)	80.7 ± 0.8 (80.0–81.8)	74.4 ± 1.8 (72.2–76.5)	80.7 ± 0.8 (80.0–81.8)	75.6 ± 1.1 (74.3–76.5)
DGO	5.3 ± 0.4 (4.5–6.0)	4.1 ± 0.4 (3.5–5.0)	5.5 ± 0.5 (5.0–6.0)	4.2 ± 0.4 (4.0–5.0)	5.1 ± 0.5 (4.5–5.5)	4.3 ± 0.5 (4.0–5.0)	4.8 ± 0.3 (3.5–4.0)	4.1 ± 0.3 (4.0–4.5)
O	10.5 ± 1.0 (8.8–12.0)	23.6 ± 2.4 (20.0–27.8)	10.4 ± 1.0 (9.1–11.7)	24.9 ± 2.8 (22.2–29.4)	9.9 ± 1.1 (8.8–11.0)	24.9 ± 3.3 (22.2–24.9)	9.2 ± 0.6 (8.8–10.0)	24.5 ± 1.6 (22.9–26.5)
Lip width	4.8 ± 0.6 (4.0–5.5)	4.0 ± 0.2 (3.5–4.5)	4.2 ± 0.4 (4.0–5.0)	3.7 ± 0.3 (3.5–4.0)	4.1 ± 0.3 (4.0–4.5)	3.8 ± 0.3 (3.5–4.0)	4.1 ± 0.3 (4.0–4.5)	3.8 ± 0.3 (3.5–4.0)
Median bulb length	22.6 ± 2.9 (17.0–26.0)	24.3 ± 1.3 (22.0–27.0)	25.4 ± 0.5 (25.0–26.0)	24.4 ± 0.9 (24.0–26.0)	25.5 ± 0.6 (25.0–26.0)	24.5 ± 1.0 (24.0–26.0)	25.5 ± 0.6 (25.0–26.0)	23.8 ± 0.5 (23.0–24.0)
Median bulb width	11.2 ± 0.8 (10.0–13.0)	7.9 ± 0.4 (7.0–8.5)	11.3 ± 1.0 (10.0–12.5)	7.8 ± 0.6 (7.0–8.5)	11.3 ± 0.8 (10.2–12.4)	7.8 ± 0.6 (7.0–8.5)	11.3 ± 1.0 (10.0–12.0)	7.5 ± 0.4 (7.0–8.0)
Anterior end to center median bulb	74.8 ± 3.1 (70.0–81.0)	48.4 ± 1.5 (46.0–51.0)	77.0 ± 3.4 (73.0–81.0)	47.8 ± 0.8 (47.0–49.0)	76.3 ± 3.4 (73.0–81.0)	47.8 ± 1.0 (47.0–49.0)	75.8 ± 2.5 (73.0–79.0)	47.8 ± 1.0 (47.0–49.0)
MB	65.9 ± 4.8 (59.5–74.0)	53.5 ± 3.2 (48.5–57.5)	64.5 ± 6.6 (59.5–76.0)	53.7 ± 3.2 (53.9–56.6)	65.5 ± 8.2 (59.5–77.5)	54.2 ± 3.2 (50.0–57.3)	65.5 ± 8.2 (59.5–77.6)	54.2 ± 3.2 (51.0–57.3)
Nerve ring to anterior end	90.9 ± 7.1 (78.0–99.0)	65.0 ± 3.2 (61.0–71.0)	95.4 ± 2.7 (91.0–98.0)	64.2 ± 2.3 (62.0–69.0)	94.8 ± 2.6 (91.0–97.0)	64.8 ± 2.2 (63.0–68.0)	94.8 ± 2.6 (91.0–97.0)	64.8 ± 2.2 (63.0–68.0)
Excretory pore to anterior end	94.6 ± 6.8 (82.0–106.0)	83.1 ± 6.0 (74.0–91.0)	98.2 ± 3.3 (94.0–103.0)	81.6 ± 5.7 (75.0–88.0)	98.5 ± 3.7 (94.0–103.0)	81.8 ± 5.6 (76.0–87.0)	98.5 ± 3.7 (94.0–103.0)	81.8 ± 5.6 (76.0–87.0)
Pharynx length	112.2 ± 12.0 (97.0–132.0)	90.8 ± 6.6 (82.0–101.0)	119.4 ± 12.1 (100.0–129.0)	89.2 ± 5.3 (83.0–97.0)	116.5 ± 13.5 (98.0–127.0)	88.3 ± 5.3 (82.0–94.0)	116.5 ± 13.5 (98.0–127.0)	88.3 ± 5.3 (82.0–94.0)
Maximum body diam.	19.4 ± 2.8 (15.0–26.0)	18.5 ± 1.3 (16.0–20.0)	21.0 ± 2.3 (19.0–25.0)	18.0 ± 1.6 (16.0–20.0)	20.5 ± 1.7 (19.0–23.0)	17.8 ± 1.7 (16.0–20.0)	20.5 ± 1.7 (19.0–23.0)	17.8 ± 1.7 (16.0–20.0)
Tail length	35.2 ± 4.2 (23.5–42.0)	39.6 ± 4.3 (31.0–44.0)	35.2 ± 6.2 (25.0–41.0)	38.2 ± 4.3 (32.0–43.0)	35.3 ± 5.9 (27.0–40.0)	37.0 ± 3.2 (33.0–40.0)	35.3 ± 5.9 (27.0–40.0)	36.0 ± 3.6 (31.0–39.0)
Anal body diam.	10.2 ± 0.8 (9.0–11.0)	12.5 ± 0.9 (11.5–14.0)	10.7 ± 0.7 (9.5–11.0)	12.6 ± 0.8 (11.5–13.5)	10.8 ± 0.5 (10.0–11.0)	12.1 ± 0.9 (11.0–13.0)	10.8 ± 0.5 (10.0–11.0)	12.1 ± 0.9 (11.0–13.0)

* Abbreviations: a = body length/greatest body diameter; b = body length/distance from anterior end to pharyngo-intestinal junction; DGO = distance between stylet base and orifice of dorsal pharyngeal gland; c = body length/tail length; c’ = tail length/tail diameter at anus or cloaca; G1 = anterior genital branch length expressed as percentage (%) of the body length; L = overall body length; m = length of conus as percentage of total stylet length; MB = distance between anterior end of body and center of median pharyngeal bulb expressed as percentage (%) of the pharynx length; n = number of specimens on which measurements are based; O = DGO as percentage of stylet length; V = distance from body anterior end to vulva expressed as percentage (%) of the body length.

**Table 7:** Morphometrics of *Paratylenchus macrodorus* Brzeski, 1963 from Santa María de Trasierra, Córdoba, Spain. All measurements are in µm and in the form: mean ± s.d. (range).

Life Stage	Females	Male	Fourth-Stage Juveniles
Sample Code	AR_102		
Locality, province	St. Mª Trasierra, Córdoba		
n	10	1	3
L	365.8.3 ± 26.6 (317–410)	395	304.3 ± 6.1 (299–311)
a *	23.0 ± 2.6 (19.9–28.3)	31.6	20.8 ± 0.6 (20.2–21.4)
b	2.8 ± 0.2 (2.5–3.0)	4.0	2.8 ± 0.1 (2.7–2.8)
c	8.8 ± 1.1 (7.4–11.1)	12.3	13.6 ± 0.5 (13.2–14.1)
c’	4.2 ± 0.5 (3.5–4.9)	2.9	2.7 ± 0.03 (2.7–2.8)
V or T	75.1 ± 0.8 (73.9–76.3)	48.1	-
G1	29.7 ± 3.1 (24.1–33.8)	-	-
Stylet length	76.2 ± 3.9 (70.0–84.0)	-	62.3 ± 1.5 (61.0–64.0)
(Stylet length/body length) × 100	20.9 ± 1.6 (18.8–24.0)	-	20.5 ± 0.3 (20.1–20.7)
Conus length	68.9 ± 4.3 (61.5–77.0)	-	53.0 ± 1.0 (52.0–54.0)
m	90.4 ± 1.6 (87.9–93.7)	-	85.0 ± 1.6 (83.9–86.9)
DGO	5.6 ± 0.6 (5.0–6.5)	-	5.5 ± 0.5 (5.0–6.0)
O	7.4 ± 0.8 (6.4–9.0)	-	8.8 ± 0.8 (8.2–8.7)
Lip width	4.7 ± 0.2 (4.5–5.0)	3.5	4.7 ± 0.6 (4.0–5.0)
Median bulb length	26.7 ± 2.4 (24.0–31.0)	-	18.7 ± 0.6 (18.0–19.0)
Median bulb width	10.3 ± 1.2 (9.0–13.0)	-	8.8 ± 0.3 (8.5–9.0)
Anterior end to center median bulb	92.9 ± 6.4 (83.0–102.0)	-	72.0 ± 1.0 (71.0–73.0)
MB	69.3 ± 3.2 (61.5–72.2)	-	66.1 ± 0.3 (65.8–66.4)
Nerve ring to anterior end	109.7 ± 10.0 (91.0–123.0)	84	85.7 ± 1.5 (84.0–87.0)
Excretory pore to anterior end	94.1 ± 9.0 (82.0–109.0)	91	73.0 ± 2.0 (71.0–75.0)
Pharynx length	133.1 ± 9.4 (115.0–143.0)	98	109.0 ± 2.0 (107.0–111.0)
Maximum body diam.	16.0 ± 1.1 (14.5–18.0)	12.5	14.7 ± 0.6 (14.0–15.0)
Tail length	42.0 ± 4.4 (33.5–49.0)	32	22.3 ± 0.6 (22.0–23.0)
Anal body diam.	10.0 ± 1.1 (8.5–12.0)	11	8.2 ± 0.3 (8.0–8.5)
Spicules	-	19.5	-
Gubernaculum	-	5.5	-

* Abbreviations: a = body length/greatest body diameter; b = body length/distance from anterior end to pharyngo-intestinal junction; DGO = distance between stylet base and orifice of dorsal pharyngeal gland; c = body length/tail length; c’ = tail length/tail diameter at anus or cloaca; G1 = anterior genital branch length expressed as percentage (%) of the body length; L = overall body length; m = length of conus as percentage of total stylet length; MB = distance between anterior end of body and center of median pharyngeal bulb expressed as percentage (%) of the pharynx length; n = number of specimens on which measurements are based; O = DGO as percentage of stylet length; T = distance from cloacal aperture to anterior end of testis expressed as percentage (%) of the body length; V = distance from body anterior end to vulva expressed as percentage (%) of the body length.

**Table 8:** Morphometrics of *Paratylenchus pandatus* (Raski, 1976) Siddiqi, 1986 from Caravaca, Murcia, Spain. All measurements are in µm and in the form: mean ± s.d. (range).

Life Stage	Females	Fourth-Stage Juveniles	Females
Sample Code	PIN_AR	PIN_AR	Type
Locality, Province	Caravaca, Murcia	Caravaca, Murcia	Population [37]
n	12	3	10
L	317.2 ± 15.9 (290–339)	277.3 ± 22.7 (252–296)	330–420
a *	18.7 ± 1.8 (15.7–21.5)	18.1 ± 1.5 (16.8–19.7)	23–32
b	2.7 ± 0.1 (2.5–2.9)	3.8 ± 0.3 (3.5–4.0)	2.8–3.2
c	12.2 ± 2.2 (9.2–16.6)	13.2 ± 1.6 (11.5–14.2)	9–12
c’	2.5 ± 0.2 (2.2–3.0)	2.2 ± 0.1 (2.1–2.3)	4.7
V	75.8 ± 0.9 (74.5–77.7)	-	70–76
G1	32.0 ± 2.9 (28.6–38.6)	-	-
Stylet length	61.3 ± 3.8 (57.0–68.5)	-	63–70
(Stylet length/body length) × 100	19.3 ± 0.8 (17.8–21.1)	-	-
Conus length	53.2 ± 3.1 (49.0–59.0)	-	-
m	86.9 ± 1.1 (85.7–89.4)	-	-
DGO	5.5 ± 0.5 (5.0–6.5)	-	-
O	9.1 ± 0.9 (7.9–10.5)	-	-
Lip width	5.9 ± 0.7 (5.0–7.0)	4.7 ± 0.3 (4.5–5.0)	-
Median bulb length	23.7 ± 2.1 (19.0–26.5)	-	-
Median bulb width	11.0 ± 1.1 (10.0–14.0)	-	-
Anterior end to center median bulb	79.5 ± 6.7 (64.0–88.0)	-	-
MB	67.0 ± 2.5 (62.8–71.3)	-	-
Nerve ring to anterior end	97.7 ± 6.3 (82.0–106.0)	55.0 ± 1.0 (54.0–56.0)	-
Excretory pore to anterior end	84.9 ± 8.9 (71.0–111.0)	63.0 ± 1.0 (62.0–64.0)	94–119
Pharynx length	118.6 ± 7.6 (102.0–128.0)	73.0 ± 1.0 (72.0–74.0)	-
Maximum body diam.	17.1 ± 1.4 (15.0–19.5)	15.3 ± 0.6 (15.0–16.0)	17
Tail length	26.7 ± 4.6 (18.0–32.0)	21.0 ± 1.0 (20.0–22.0)	38
Anal body diam.	10.6 ± 1.5 (8.0–13.0)	9.3 ± 0.3 (9.0–9.5)	-

* Abbreviations: a = body length/greatest body diameter; b = body length/distance from anterior end to pharyngo-intestinal junction; DGO = distance between stylet base and orifice of dorsal pharyngeal gland; c = body length/tail length; c’ = tail length/tail diameter at anus or cloaca; G1 = anterior genital branch length expressed as percentage (%) of the body length; L = overall body length; m = length of conus as percentage of total stylet length; MB = distance between anterior end of body and center of median pharyngeal bulb expressed as percentage (%) of the pharynx length; n = number of specimens on which measurements are based; O = DGO as percentage of stylet length; V = distance from body anterior end to vulva expressed as percentage (%) of the body length.

**Table 9:** Morphometrics of *Paratylenchus recisus* Siddiqi, 1996 from Arroyo Frío, Jaén, Spain. All measurements are in µm and in the form: mean ± s.d. (range).

Life Stage	Females	Fourth-Stage Juveniles	Females
Sample Code	CAZ_06	CAZ_06	Type
Locality, Province	Arroyo Frío, Jaén	Arroyo Frío, Jaén	Population [43]
n	4	3	20
L	397.0 ± 36.5 (363–448)	353.0 ± 32.5 (329–390)	270–390
a *	18.4 ± 1.3 (17.2–20.2)	16.2 ± 1.0 (15.5–17.3)	16–27
b	4.6 ± 0.7 (3.8–5.4)	4.9 ± 0.3 (4.7–5.3)	3.8–5.0
c	12.6 ± 1.0 (11.2–13.6)	14.7 ± 0.8 (14.2–15.6)	13–16
c’	3.3 ± 0.4 (2.8–3.5)	2.4 ± 0.1 (2.4–2.6)	2.7–3.3
V	81.2 ± 0.7 (80.4–82.0)	-	78–83
G1	44.3 ± 1.6 (42.1–45.8)	-	-
Stylet length	15.1 ± 0.6 (14.5–16.0)	-	15–17
(Stylet length/body length) × 100	3.8 ± 0.4 (3.3–4.2)	-	-
Conus length	9.8 ± 0.5 (9.0–10.0)	-	-
m	64.5 ± 2.5 (62.1–66.7)	-	-
DGO	5.3 ± 0.5 (5.0–6.0)	-	-
O	34.8 ± 4.5 (31.3–41.4)	-	-
Lip width	5.6 ± 0.8 (5.0–6.5)	4.5 ± 0.5 (4.0–5.0)	-
Median bulb length	23.0 ± 2.2 (20.0–25.0)	14.0 ± 0.5 (13.5–14.5)	-
Median bulb width	10.4 ± 1.5 (9.0–12.5)	7.2 ± 0.3 (7.0–7.5)	-
Anterior end to center median bulb	47.5 ± 7.4 (39.0–57.0)	33.7 ± 1.5 (32.0–35.0)	-
MB	54.0 ± 4.9 (47.0–58.0)	46.7 ± 0.9 (45.7–47.3)	-
Nerve ring to anterior end	66.3 ± 7.1 (59.0–76.0)	62.0 ± 2.0 (60.0–64.0)	-
Excretory pore to anterior end	77.5 ± 9.7 (71.0–92.0)	68.7 ± 1.5 (67.0–70.0)	58–70
Pharynx length	87.8 ± 9.1 (81.0–101.0)	72.0 ± 2.0 (70.0–74.0)	-
Maximum body diam.	21.8 ± 3.3 (18.0–26.0)	21.8 ± 0.8 (21.0–22.5)	12–16
Tail length	31.5 ± 2.6 (28.0–34.0)	24.0 ± 1.0 (23.0–25.0)	18–29
Anal body diam.	9.8 ± 1.7 (8.0–12.0)	9.8 ± 0.8 (9.0–10.5)	-

* Abbreviations: a = body length/greatest body diameter; b = body length/distance from anterior end to pharyngo-intestinal junction; DGO = distance between stylet base and orifice of dorsal pharyngeal gland; c = body length/tail length; c’ = tail length/tail diameter at anus or cloaca; G1 = anterior genital branch length expressed as percentage (%) of the body length; L = overall body length; m = length of conus as percentage of total stylet length; MB = distance between anterior end of body and center of median pharyngeal bulb expressed as percentage (%) of the pharynx length; n = number of specimens on which measurements are based; O = DGO as percentage of stylet length; V = distance from body anterior end to vulva expressed as percentage (%) of the body length.

**Table 10:** Morphometrics of *Paratylenchus sheri* (Raski, 1973) Siddiqi, 1986 from Arroyo Frío and Coto Ríos, Jaén, Spain. All measurements are in µm and in the form: mean ± s.d. (range).

Life Stage	Females	Fourth-Stage Juveniles	Females
Sample Code	CAZ_04	CAZ_04	CAZ_07
Locality, Province	Arroyo Frío, Jaén	Arroyo Frío, Jaén	Coto Ríos, Jaén
n	14	5	4
L	548.6 ± 53.2 (459–626)	523.6 ± 11.6 (514–543)	540.0 ± 45.4 (492–595)
a *	20.5 ± 2.5 (15.8–24.6)	23.6 ± 1.3 (21.5–24.8)	19.9 ± 2.3 (16.6–22.0)
b	4.8 ± 0.5 (3.8–5.4)	4.9 ± 0.2 (4.6–5.1)	4.8 ± 0.3 (4.6–5.2)
c	11.1 ± 1.6 (9.2–14.6)	14.3 ± 2.9 (11.1–17.9)	11.5 ± 1.5 (10.3–13.5)
c’	3.8 ± 0.3 (3.2–4.2)	3.0 ± 0.2 (2.8–3.3)	3.6 ± 0.3 (3.2–3.9)
V	78.9 ± 1.7 (75.8–81.7)	-	79.5 ± 1.7 (77.0–80.9)
G1	49.7 ± 5.6 (40.7–56.7)	-	48.5 ± 7.0 (41.3–55.0)
Stylet length	23.8 ± 0.7 (22.5–25.0)	20.0 ± 0.4 (19.5–20.5)	23.6 ± 0.5 (23.0–24.0)
(Stylet length/body length) × 100	4.4 ± 0.4 (4.0–5.5)	3.9 ± 0.1 (3.8–4.0)	4.4 ± 0.4 (4.0–4.9)
Conus length	15.4 ± 0.8 (14.0–17.0)	12.3 ± 0.3 (12.0–12.5)	14.8 ± 0.5 (14.0–15.0)
m	64.5 ± 2.5 (62.0–70.8)	61.5 ± 1.1 (60.0–62.5)	62.4 ± 1.2 (60.9–63.8)
DGO	6.0 ± 0.5 (5.0–7.0)	4.2 ± 0.4 (4.0–5.0)	6.3 ± 0.5 (6.0–7.0)
O	25.3 ± 2.1 (20.8–30.4)	21.0 ± 1.9 (20.0–24.4)	26.5 ± 2.6 (25.0–30.4)
Lip width	5.1 ± 0.6 (4.5–6.0)	3.9 ± 0.4 (3.5–4.5)	5.0 ± 0.7 (4.5–6.0)
Median bulb length	27.2 ± 1.9 (24.0–31.0)	-	25.8 ± 1.3 (24.0–27.0)
Median bulb width	14.3 ± 1.9 (12.0–18.5)	-	13.3 ± 1.2 (12.0–14.0)
Anterior end to center median bulb	63.0 ± 3.8 (56.0–70.0)	-	61.8 ± 3.9 (57.0–65.0)
MB	55.6 ± 1.5 (52.5–58.1)	-	55.2 ± 1.0 (54.3–56.5)
Nerve ring to anterior end	86.0 ± 6.4 (76.0–99.0)	82.4 ± 1.7 (81.0–85.0)	83.3 ± 6.8 (76.0–90.0)
Excretory pore to anterior end	101.9 ± 6.4 (93.0–116.0)	99.2 ± 1.9 (97.0–102.0)	100.8 ± 6.9 (95.0–110.0)
Pharynx length	113.4 ± 6.3 (104.0–126.0)	107.0 ± 5.0 (102.0–115.0)	111.8 ± 6.4 (105.0–119.0)
Maximum body diam.	27.1 ± 4.0 (21.0–33.5)	22.2 ± 1.3 (21.0–24.0)	27.3 ± 2.7 (24.5–31.0)
Tail length	50.1 ± 7.0 (39.0–62.0)	38.0 ± 8.3 (29.0–49.0)	47.5 ± 5.5 (42.0–54.0)
Anal body diam.	13.3 ± 1.2 (11.5–15.5)	12.8 ± 2.1 (10.5–15.0)	13.3 ± 1.0 (12.0–14.0)

* Abbreviations: a = body length/greatest body diameter; b = body length/distance from anterior end to pharyngo-intestinal junction; DGO = distance between stylet base and orifice of dorsal pharyngeal gland; c = body length/tail length; c’ = tail length/tail diameter at anus or cloaca; G1 = anterior genital branch length expressed as percentage (%) of the body length; L = overall body length; m = length of conus as percentage of total stylet length; MB = distance between anterior end of body and center of median pharyngeal bulb expressed as percentage (%) of the pharynx length; n = number of specimens on which measurements are based; O = DGO as percentage of stylet length; V = distance from body anterior end to vulva expressed as percentage (%) of the body length.

**Table 11:** Morphometrics of *Paratylenchus variabilis* Raski, 1975 from Córdoba, Córdoba, Spain. All measurements are in µm and in the form: mean ± s.d. (range).

Life Stage	Females	Fourth-Stage Juveniles	Females
Sample Code	EP_ACA	EP_ACA	Type
Locality, Province	Córdoba, Córdoba	Córdoba, Córdoba	Population [46]
n	10	4	27
L	337.3 ± 30.5 (302–407)	312.3 ± 38.2 (282–367)	250–340
a *	21.7 ± 1.1 (20.1–23.5)	21.2 ± 2.3 (19.4–24.5)	19–25
b	3.9 ± 0.6 (2.8–4.8)	3.9 ± 0.2 (3.7–4.2)	3.6–4.7
c	15.3 ± 1.2 (13.1–17.2)	14.7 ± 1.3 (13.1–16.3)	12–18
c’	2.6 ± 0.2 (2.3–2.9)	2.2 ± 0.2 (1.9–2.4)	2.7
V	84.5 ± 1.0 (83.1–86.5)	-	82–85
G1	39.4 ± 6.6 (28.6–52.8)	-	-
Stylet length	14.9 ± 0.6 (14.0–16.0)	11.6 ± 0.8 (11.0–12.5)	13–16
(Stylet length/body length) × 100	4.5 ± 0.4 (3.4–4.9)	3.7 ± 0.3 (3.3–4.0)	-
Conus length	10.5 ± 0.5 (10.0–11.0)	7.5 ± 0.4 (7.0–8.0)	-
m	70.5 ± 3.7 (64.5–75.9)	64.6 ± 2.5 (62.5–68.2)	-
DGO	3.5 ± 0.6 (2.5–4.5)	2.6 ± 0.5 (2.0–3.0)	-
O	23.5 ± 3.9 (17.9–30.0)	22.6 ± 4.2 (18.2–27.3)	-
Lip width	5.6 ± 0.6 (5.0–6.5)	4.3 ± 0.3 (4.0–4.5)	-
Median bulb length	18.9 ± 1.7 (17.0–23.0)	18.0 ± 1.4 (17.0–20.0)	-
Median bulb width	8.9 ± 1.4 (7.5–12.0)	7.5 ± 0.6 (7.0–8.0)	-
Anterior end to center median bulb	46.0 ± 3.6 (40.0–52.0)	41.0 ± 0.8 (40.0–42.0)	-
MB	53.1 ± 7.4 (39.5–63.6)	51.9 ± 3.6 (46.6–54.5)	-
Nerve ring to anterior end	62.6 ± 3.0 (58.0–68.0)	57.8 ± 5.0 (51.0–63.0)	-
Excretory pore to anterior end	74.5 ± 7.8 (67.0–95.0)	67.0 ± 6.2 (60.0–75.0)	59–71
Pharynx length	87.9 ± 12.4 (77.0–119.0)	79.3 ± 5.9 (76.0–88.0)	-
Maximum body diam.	15.6 ± 1.6 (14.0–19.0)	14.8 ± 0.6 (14.0–15.5)	13
Tail length	22.2 ± 3.5 (20.0–31.0)	21.4 ± 2.6 (19.0–25.0)	21
Anal body diam.	8.8 ± 1.8 (7.0–13.0)	9.8 ± 1.0 (9.0–11.0)	-

* Abbreviations: a = body length/greatest body diameter; b = body length/distance from anterior end to pharyngo-intestinal junction; DGO = distance between stylet base and orifice of dorsal pharyngeal gland; c = body length/tail length; c’ = tail length/tail diameter at anus or cloaca; G1 = anterior genital branch length expressed as percentage (%) of the body length; L = overall body length; m = length of conus as percentage of total stylet length; MB = distance between anterior end of body and center of median pharyngeal bulb expressed as percentage (%) of the pharynx length; n = number of specimens on which measurements are based; O = DGO as percentage of stylet length; V = distance from body anterior end to vulva expressed as percentage (%) of the body length.

**Table 12:** Morphometrics of *Paratylenchus verus* (Brzeski, 1995) Brzeski, 1998 from Santa María de Trasierra, Córdoba, Spain. All measurements are in µm and in the form: mean ± s.d. (range).

	Females	Fourth-Stage Juveniles	Females
Sample Code	AR_097	AR_097	[28]
Locality	Sta. Mª Trasierra, Córdoba	Sta. Mª Trasierra, Córdoba	Type Population
n	14	7	11
L	324.4 ± 25.0 (265–355)	276.9 ± 22.2 (253–319)	270–320
a *	19.4 ± 3.7 (12.2–24.7)	21.8 ± 2.3 (18.8–24.2)	20–24
b	2.4 ± 0.2 (2.1–2.7)	3.0 ± 0.5 (2.5–3.8)	2.2–2.5
c	12.1 ± 2.0 (9.1–16.9)	11.1 ± 1.1 (9.4–12.5)	13–19
c’	3.3 ± 0.3 (2.7–3.6)	3.0 ± 0.3 (2.8–3.3)	1.8–2.9
V	77.0 ± 1.8 (73.6–80.3)	-	76–79
G1	29.3 ± 4.1 (24.5–40.4)	-	-
Stylet length	89.1 ± 5.8 (79.0–97.0)	49.1 ± 1.2 (48.0–51.0)	66–86
(Stylet length/body length) × 100	27.6 ± 1.9 (24.8–30.2)	17.8 ± 1.4 (15.8–20.2)	-
Conus length	77.8 ± 5.3 (70.0–86.5)	41.9 ± 1.2 (40.0–43.0)	-
m	87.3 ± 1.9 (84.3–89.9)	85.2 ± 1.1 (83.3–86.9)	-
DGO	4.6 ± 0.7 (3.5–6.0)	4.2 ± 0.3 (4.0–4.5)	-
O	5.2 ± 0.8 (4.1–6.7)	8.6 ± 0.6 (7.8–9.4)	-
Lip width	4.4 ± 0.4 (4.0–5.0)	3.7 ± 0.3 (3.5–4.0)	-
Median bulb length	25.5 ± 3.3 (22.0–34.0)	15.2 ± 1.2 (14.0–17.0)	-
Median bulb width	10.6 ± 0.7 (9.5–12.0)	8.3 ± 0.4 (8.0–9.0)	-
Anterior end to center median bulb	101.7 ± 7.9 (87.0–115.0)	69.3 ± 3.4 (66.0–74.0)	-
MB	76.1 ± 4.0 (70.2–83.0)	73.7 ± 5.3 (68.3–83.5)	-
Nerve ring to anterior end	112.6 ± 11.3 (94.0–133.0)	77.9 ± 0.9 (77.0–79.0)	-
Excretory pore to anterior end	89.0 ± 5.2 (81.0–95.0)	82.0 ± 2.5 (78.0–85.0)	68–92
Pharynx length	133.9 ± 12.1 (112.0–161.0)	94.4 ± 7.8 (85.0–105.0)	-
Maximum body diam.	17.4 ± 4.2 (13.0–27.0)	12.9 ± 2.3 (11.0–17.0)	14
Tail length	27.1 ± 2.6 (21.0–30.0)	25.3 ± 3.9 (23.0–34.0)	18
Anal body diam.	8.1 ± 0.5 (7.0–9.0)	8.4 ± 1.4 (7.5–11.5)	-

* Abbreviations: a = body length/greatest body diameter; b = body length/distance from anterior end to pharyngo-intestinal junction; DGO = distance between stylet base and orifice of dorsal pharyngeal gland; c = body length/tail length; c’ = tail length/tail diameter at anus or cloaca; G1 = anterior genital branch length expressed as percentage (%) of the body length; L = overall body length; m = length of conus as percentage of total stylet length; MB = distance between anterior end of body and center of median pharyngeal bulb expressed as percentage (%) of the pharynx length; n = number of specimens on which measurements are based; O = DGO as percentage of stylet length; V = distance from body anterior end to vulva expressed as percentage (%) of the body length.

**Table 13:** Morphometrics of *Paratylenchus vitecus* (Pramodini et al., 2006) Ghaderi et al., 2014 from Córdoba, Córdoba, Spain. All measurements are in µm and in the form: mean ± s.d. (range).

	Females	Fourth-Stage Juveniles	Females
Sample Code	EP_ACA	EP_ACA	Type Population
Locality	Córdoba, Córdoba	Córdoba, Córdoba	[47]
n	3	7	7
L	341.2 ± 22.1 (323–366)	271.6 ± 12.0 (259–288)	220–350
a *	20.3 ± 1.7 (18.3–21.5)	19.5 ± 1.4 (18.8–24.2)	20–26
b	2.7 ± 0.1 (2.5–2.8)	2.9 ± 0.2 (2.6–3.1)	2.7–2.9
c	11.6 ± 1.6 (9.8–12.9)	10.6 ± 0.6 (9.6–11.5)	9–16
c’	3.1 ± 0.4 (2.7–3.5)	2.9 ± 0.2 (2.7–3.1)	2.9
V	72.8.0 ± 4.1 (68.1–75.4)	-	72–77
G1	28.3 ± 3.8 (24.5–32.1)	-	-
Stylet length	66.0 ± 4.0 (62.0–70.0)	42.5 ± 1.6 (40.0–44.0)	42–65
(Stylet length/body length) × 100	19.4 ± 2.1 (16.9–20.8)	15.7 ± 1.1 (13.9–16.7)	-
Conus length	59.3 ± 1.5 (58.0–61.0)	35.6 ± 1.0 (34.0–37.0)	-
m	90.0 ± 3.2 (87.1–93.5)	83.7 ± 1.5 (81.8–85.7)	-
DGO	4.3 ± 0.6 (4.0–5.0)	3.1 ± 0.4 (2.5–4.0)	-
O	6.1 ± 0.8 (5.4–7.0)	7.2 ± 1.1 (6.3–9.5)	-
Lip width	4.5 ± 0.5 (4.0–5.0)	4.5 ± 0.5 (4.0–5.0)	-
Median bulb length	24.0 ± 2.0 (22.0–26.0)	17.6 ± 3.8 (15.0–26.0)	-
Median bulb width	9.3 ± 2.3 (8.0–12.0)	8.1 ± 0.6 (7.5–9.0)	-
Anterior end to center median bulb	88.3 ± 4.9 (85.0–94.0)	57.1 ± 3.1 (53.0–61.0)	-
MB	68.9 ± 1.8 (67.2–70.8)	59.9 ± 0.9 (58.2–61.1)	-
Nerve ring to anterior end	105.7 ± 7.2 (101.0–114.0)	70.0 ± 3.7 (63.0–74.0)	-
Excretory pore to anterior end	84.3 ± 6.8 (79.0–92.0)	78.4 ± 6.6 (72.0–89.0)	85–94
Pharynx length	128.3 ± 8.5 (120.0–137.0)	95.4 ± 4.4 (91.0–102.0)	-
Maximum body diam.	17.0 ± 2.6 (15.0–20.0)	14.0 ± 1.6 (12.5–16.5)	10–15
Tail length	29.8 ± 3.5 (26.0–33.0)	25.6 ± 1.5 (24.0–27.5)	14–39
Anal body diam.	9.8 ± 1.5 (8.5–11.5)	8.8 ± 0.6 (8.0–9.5)	-

* Abbreviations: a = body length/greatest body diameter; b = body length/distance from anterior end to pharyngo-intestinal junction; DGO = distance between stylet base and orifice of dorsal pharyngeal gland; c = body length/tail length; c’ = tail length/tail diameter at anus or cloaca; G1 = anterior genital branch length expressed as percentage (%) of the body length; L = overall body length; m = length of conus as percentage of total stylet length; MB = distance between anterior end of body and center of median pharyngeal bulb expressed as percentage (%) of the pharynx length; n = number of specimens on which measurements are based; O = DGO as percentage of stylet length; V = distance from body anterior end to vulva expressed as percentage (%) of the body length.

## Data Availability

The datasets generated during and/or analyzed during the current study are available from the corresponding author on reasonable request.

## References

[1] Micoletzky H. (1922). Die freilebenden Erd-Nematoden. Arch. Für Nat..

[2] Ghaderi R., Geraert E., Karegar A. (2016). The Tylenchulidae of the World, Identification of the Family Tylenchulidae (Nematoda: Tylenchida).

[3] Singh R., Karssen G., Coureur M., Subbotin S., Bert W. (2021). Integrative taxonomy and molecular phylogeny of the plant-parasitic nematode genus *Paratylenchus* (Nematoda: Paratylenchinae): Linking species with molecular barcodes. Plants.

[4] Clavero-Camacho I., Cantalapiedra-Navarrete C., Archidona-Yuste A., Castillo P., Palomares-Rius J.E. (2021). Remarkable cryptic diversity of *Paratylenchus* spp. (Nematoda: Tylenchulidae) in Spain. Animals.

[5] Raski D.J. (1962). Paratylenchidae n.fam. with descriptions of five new species of *Gracilacus* n.g. and an emendation of *Cacopaurus* Thorne, 1943, *Paratylenchus* Micoletzky, 1922 and Criconematidae Thorne, 1943. Proc. Helminthol. Soc. Wash..

[6] Van den Berg E., Tiedt L.R., Subbotin S.A. (2014). Morphological and molecular characterisation of several *Paratylenchus* Micoletzky, 1922 (Tylenchida: Paratylenchidae) species from South Africa and USA, together with some taxonomic notes. Nematology.

[7] Munawar M., Miao W., Castillo P., Zheng J.-W. (2020). A new pin nematode, *Paratylenchus sinensis* n. sp. (Nematoda: Paratylenchinae) in the rhizosphere of white mulberry from Zhejiang Province, China. Eur. J. Plant Pathol..

[8] Munawar M., Yevtushenko D.P., Palomares-Rius J.E., Castillo P. (2021). Species diversity of pin nematodes (*Paratylenchus* spp.) from potato growing regions of southern Alberta, Canada. Plants.

[9] Castillo P., Gómez-Barcina A. (1988). Some species of Tylenchida from natural habitats in southeastern Spain. Nematol. Medit..

[10] Akyazi F., Felek A.F., Čermák V., Čudejková M., Foit J., Yildiz S., Háněl L. (2015). Description of *Paratylenchus* (*Gracilacus*) *straeleni* (De Coninck, 1931) Oostenbrink, 1960 (Nematoda: Criconematoidea, Tylenchulidae) from hazelnut in Turkey and its comparison with other world populations. Helminthologia.

[11] Powers T.O., Harris T.S., Higgins R.S., Mullin P.G., Powers K.S. (2020). Nematode biodiversity assessments need vouchered databases: A BOLD reference library for plant-parasitic nematodes in the superfamily Criconematoidea. Genome.

[12] Hernandez M., Mateo M.D., Jordana R. (1988). Estudio comparativo entre grupos tróficos de Nematodos del suelo de cinco bosques de Navarra (tres naturales y dos de repoblación). Actas II Congr. Mund. Vasco. Sec. Biol. Ambient..

[13] Brzeski M., Hanel L., Nico A., Castillo P. (1999). Paratylenchinae: Redescription of *Paratylenchus arculatus* Luc & de Guiran, 1962, a new senior synonym of *P. nainianus* Edward & Misra, 1963 (Nematoda: Tylenchulidae). Nematology.

[14] Nico A.I., Rapoport H.F., Jiménez-Díaz R.M., Castillo P. (2002). Incidence and population density of plant-parasitic nematodes associated with olive planting stocks at nurseries in southern Spain. Plant Dis..

[15] Peña-Santiago R. (1990). Plant-parasitic nematodes associated with olive (*Olea europea* L.) in the province of Jaén, Spain. Rev. Nématol..

[16] Archidona-Yuste A., Wiegand T., Castillo P., Navas-Cortés J.A. (2019). Dataset on the diversity of plant-parasitic nematodes in cultivated olive trees in southern Spain. Data Brief.

[17] Gomez-Barcina A., Castillo P., Pais M.A.G. (1990). Four species of the genus *Paratylenchus* Micoletzky from southeasthern Spain. Nematol. Medit..

[18] Talavera M., Navas A. (2002). Incidence of plant-parasitic nematodes in natural and semi-natural mountain grassland and the host status of some common grass species. Nematology.

[19] Escuer M. (1995). Els Nematodes. El Patrimoni Biològic del Montseny. Catàleg de Flora i Fauna.

[20] Castillo P., González-País M.A., Gómez-Barcina A. (1989). El género *Gracilacus* Raski, 1962 en España (Paratylenchinae: Tylenchida). Rev. Ibér. Parasitol..

[21] Gomez-Barcina A., Castillo P., González-País M.A. (1989). Nematodos fitoparásitos de la subfamilia Criconematinae Taylor, 1936 en la Sierra de Cazorla. Rev. Ibér. Parasitol..

[22] Talavera M., Tobar Jimenez A. (1997). Plant parasitic nematodes from unirrigated fields in Alhama, southeastern Spain. Nematol. Medit..

[23] Imaz A., Hernández M.A., Ariño A.H., Armendáriz I., Jordana R. (2002). Diversity of soil nematodes across a Mediterranean ecotone. Appl. Soil Ecol..

[24] Peña Santiago R., Geraert E. (1990). New data on *Aorolaimus perscitus* (Doucet, 1980) and *Gracilacus teres* Raski, 1976 (Nematoda: Tylenchida) associated with olive (*Olea europea* L.) in the province of Jaén, Spain. Nematologica.

[25] Brzeski M.W. (1977). Seasonal dynamics of *Paratylenchus bukowinensis* Micol. and some other nematodes. Rocz. Nauk Rol. Ser. E.

[26] Brzeski M.W., Hanel L. (1999). Paratylenchinae: Postembryonic developmental stages of *Paratylenchus straeleni* (De Coninck, 1931) and *P. steineri* Golden, 1961 (Nematoda: Tylenchulidae). Nematology.

[27] De Coninck L.A.P. (1931). Sur trois espèces nouvelles de nématodes libres trouvés en Belgique. Bull. Musée R. d’Hist. Nat. Belg..

[28] Brzeski M.W. (1995). Paratylenchinae: Morphology of some known species and descriptions of *Gracilacus Bilineata* sp. n. and *G. Vera* sp. n. (Nematoda: Tylenchulidae). Nematologica.

[29] Brzeski M.W. (1998). Nematodes of Tylenchina in Poland and Temperate Europe.

[30] Ghaderi R., Karegar A. (2013). Some species of *Paratylenchus* (Nematoda: Tylenchulidae) from Iran. Iran. J. Plant Pathol..

[31] Brown G.L. (1959). Three new species of the genus *Paratylenchus* from Canada (Nematoda: Criconematidae). Proc. Helminthol. Soc. Wash..

[32] Bernard E.C. (1982). Criconematina (nematoda: Tylenchida) from the aleutian islands. J. Nematol..

[33] Castillo P., Gomez-Barcina A. (1993). Plant-parasitic nematodes associated with tropical and subtropical crops in southern Spain. Nematol. Medit..

[34] Escuer M., Cano A., Bello A. (2004). Nematodos fitoparásitos de la Región de Murcia y alternativas de control. Desinfección de Suelos en Invernaderos de Pimientos.

[35] Geraert E. (1965). The Genus *Paratylenchus*. Nematologica.

[36] Oostenbrink M. (1953). A note on *Paratylenchus* in the Netherlands with the description of *P. goodeyi* n. sp. (Nematoda, Criconematidae). Tijdschr. Plantenziekten.

[37] Raski D.J. (1976). Revision of the genus *Paratylenchus* Micoletzky, 1922 and descriptions of new species. Part III of three parts—*Gracilacus*. J. Nematol..

[38] Yu Q., Ye W., Powers T. (2016). Morphological and molecular characterization of *Gracilacus wuae* n. sp. (Nematoda: Criconematoidea) associated with cow parsnip (*Heracleum maximum*) in Ontario, Canada. J. Nematol..

[39] Brzeski M. (1963). Paratylenchus macrodorus n. sp. (Nematoda, Paratylenchidae), a new plant parasitic nematode from Poland. Bull. L’Acad. Pol. Sci..

[40] Van den Berg E., Quénéhervé P., Tiedt L. (2006). Two *Paratylenchus* species (Nemata: Tylenchulidae) from Martinique and New Caledonia. J. Nematode Morphol. Syst..

[41] Nguyen C.N., Baldwin J.G., Choi Y.E. (2004). New records of *Paratylenchus* Micoletzky, 1922 (Nematoda: Paratylenchinae) from Viet Nam with description of *Paratylenchus lapcaiensis* sp. n. J. Nematode Morphol. Syst..

[42] Van Den Berg E., Mekete T., Tiedt L.R. (2004). New records of Criconematidae (Nemata) from Ethiopia. J. Nematode Morphol. Syst..

[43] Siddiqi M. (1996). *Paratylenchus recisus* sp.n. and *P. perminimus* sp.n. (Criconematina: Paratylenchidae). Afro-Asian J. Nematol..

[44] Raski D.J. (1973). *Paratylenchoides* gen. n. and two new species (Nematoda: Paratyleiichidae). Proc. Helminthol. Soc. Wash..

[45] Mirbabaei H., Eskandari A., Ghaderi R., Karegar A. (2019). On the synonymy of *Trophotylenchulus asoensis* and *T. okamotoi* with *T. arenarius*, and intra-generic structure of *Paratylenchus* (Nematoda: Tylenchulidae). J. Nematol..

[46] Raski D.J. (1975). Revision of the genus *Paratylenchus* Micoletzky, 1922 and descriptions of new species. Part I of three parts. J. Nematol..

[47] Pramodini M., Mohilal N., Dhanachand C. (2006). *Gracilacus vitecus* sp.n. and record of *G. raskii* Phukan & Sanwal from Manipur, India. Ind. J. Nematol..

[48] Siddiqi M.R. (1986). Tylenchida: Parasites of Plants and Insects.

[49] Raski D., Luc M. (1987). A reappraisal of Tylenchina (Nemata): 10. The superfamily Criconematoidea Taylor, 1936. Rev. Nématol..

[50] Derycke S., Backeljau T., Moens T. (2013). Dispersal and gene flow in free-living marine nematodes. Front. Zool..

[51] Jex A.R., Littlewood D.T., Gasser R.B. (2010). Toward next-generation sequencing of mitochondrial genomes-focus on parasitic worms of animals and biotechnological implications. Biotechnol. Adv..

[52] Coolen W.A., Lamberti F., Taylor C.E. (1979). Methods for extraction of *Meloidogyne* spp. and other nematodes from roots and soil. Root-Knot Nematodes (Meloidogyne Species): Systematics, Biology and Control.

[53] Seinhorst J.W. (1966). Killing nematodes for taxonomic study with hot F.A. 4:1. Nematologica.

[54] De Grisse A.T. (1969). Redescription ou modifications de quelques techniques utilisées dans l’étude de nématodes phytoparasitaires. Meded. Rijksfac. Landbouwwet. Gent.

[55] Hunt D.J., Palomares-Rius J.E. (2012). General morphology and morphometries of plant-parasitic nematodes. Practical Plant Nematology.

[56] Wergin W.P., Zuckerman B.M., Rohde R.A. (1981). Scanning electron microscopic techniques and applications for use in nematology. Plant Parasitic Nematodes.

[57] Palomares-Rius J.E., Clavero-Camacho I., Archidona-Yuste A., Cantalapiedra-Navarrete C., León-Ropero G., Braun Miyara S., Karssen G., Castillo P. (2021). Global distribution of the reniform nematode genus *Rotylenchulus* with the synonymy of *Rotylenchulus macrosoma* with *Rotylenchulus borealis*. Plants.

[58] De Ley P., Felix M.A., Frisse L., Nadler S., Sternberg P., Thomas W.K. (1999). Molecular and morphological characterisation of two reproductively isolated species with mirror-image anatomy (Nematoda: Cephalobidae). Nematology.

[59] Subbotin S.A., Vierstraete A., De Ley P., Rowe J., Waeyenberge L., Moens M., Vanfleteren J.R. (2001). Phylogenetic relationships within the cyst-forming nematodes (Nematoda, Heteroderidae) based on analysis of sequences from the ITS regions of ribosomal DNA. Mol. Phylogenet. Evol..

[60] Bowles J., Blair D., McManus D.P. (1992). Genetic variants within the genus *Echinococcus* identified by mitochondrial DNA sequencing. Mol. Biochem. Parasitol..

[61] Altschul S.F., Gish W., Miller W., Myers E.W., Lipman D.J. (1990). Basic local alignment search tool. J. Mol. Biol..

[62] Subbotin S.A., Yan G., Kantor M., Handoo Z. (2020). On the molecular identity of *Paratylenchus nanus* Cobb, 1923 (Nematoda: Tylenchida). J. Nematol..

[63] Katoh K., Rozewicki J., Yamada K.D. (2019). MAFFT online service: Multiple sequence alignment, interactive sequence choice and visualization. Brief. Bioinform..

[64] Hall T.A. (1999). BioEdit: A user-friendly biological sequence alignment editor and analysis program for Windows 95/98/NT. Nucleic Acids Symp. Ser..

[65] Castresana J. (2000). Selection of conserved blocks from multiple alignments for their use in phylogenetic analysis. Mol. Biol. Evol..

[66] Ronquist F., Huelsenbeck J.P. (2003). MrBayes 3: Bayesian phylogenetic inference under mixed models. Bioinformatics.

[67] Darriba D., Taboada G.L., Doallo R., Posada D. (2012). jModelTest 2: More models, new heuristics and parallel computing. Nat. Methods.

[68] Rambaut A. FigTree v1.4.2, A Graphical Viewer of Phylogenetic Trees. http://tree.bio.ed.ac.uk/software/figtree/.

